# CREBH Determines the Severity of Sulpyrine-Induced Fatal Shock

**DOI:** 10.1371/journal.pone.0055800

**Published:** 2013-02-07

**Authors:** Naganori Kamiyama, Masahiro Yamamoto, Hiroyuki Saiga, Ji Su Ma, Jun Ohshima, Sakaaki Machimura, Miwa Sasai, Taishi Kimura, Yoshiyasu Ueda, Hisako Kayama, Kiyoshi Takeda

**Affiliations:** 1 Department of Microbiology and Immunology, Graduate School of Medicine, Osaka University, Suita, Osaka, Japan; 2 Laboratory of Mucosal Immunology, WPI Immunology Frontier Research Center, Osaka University, Suita, Osaka, Japan; 3 Laboratory of Immunoparasitology, WPI Immunology Frontier Research Center, Osaka University, Suita, Osaka, Japan; 4 Department of Immunoparasitology, Research Institute for Microbial Diseases, Osaka University, Suita, Osaka, Japan; 5 Core Research for Evolutional Science and Technology, Japan Science and Technology Agency, Kawaguchi, Saitama, Japan; Beckman Research Institute of City of Hope, United States of America

## Abstract

Although the pyrazolone derivative sulpyrine is widely used as an antipyretic analgesic drug, side effects, including fatal shock, have been reported. However, the molecular mechanism underlying such a severe side effect is largely unclear. Here, we report that the transcription factor CREBH that is highly expressed in the liver plays an important role in fatal shock induced by sulpyrine in mice. CREBH-deficient mice were resistant to experimental fatal sulpyrine shock. We found that sulpyrine-induced expression of cytochrome P450 2B (CYP2B) family genes, which are involved in sulpyrine metabolism, in the liver was severely impaired in CREBH-deficient mice. Moreover, introduction of CYP2B in CREBH-deficient liver restored susceptibility to sulpyrine. Furthermore, ectopic expression of CREBH up-regulated CYP2B10 promoter activity, and *in vivo* knockdown of CREBH in wild-type mice conferred a significant resistance to fatal sulpyrine shock. These data demonstrate that CREBH is a positive regulator of CYP2B in response to sulpyrine administration, which possibly results in fatal shock.

## Introduction

Endoplasmic reticulum (ER) is a cytoplasmic organelle, which plays an important role in folding and assembly of newly synthesized proteins [1]. Accumulation of misfolded or unfolded proteins in ER induces ER stress and results in refolding or degradation of the proteins, which is termed unfolded protein response (UPR). The UPR has been shown to involve three major pathways dependent on ATF6α, IRE1α/β-XBP1, or PERK [2]. IRE1α and IRE1β are ER-localizing endonucleases, which catalyze the splicing of the XBP1 mRNA resulting in the frame shift and production of the active form of XBP1 inducing various proteins involved in UPR [3]. PERK is a kinase that phosphorylates eIF2a, leading to translational inhibition to reduce global protein loading [4]. The ATF6α-dependent pathway is regulated by the transcription factor ATF6α, which is localized in ER in unstimulated conditions [5]. In response to ER stress, ATF6α moves from ER to the Golgi apparatus, where it is cleaved by site-specific proteases, S1P and S2P, liberating the N-terminal basic leucine zipper (b-ZIP) and the transcription activation domains to the nucleus. The translocation of the N-terminus of ATF6α induces transcription of UPR-related genes that promote protein folding and degradation.

Although the three major UPR systems are shown to function ubiquitously, recent studies indicate that tissue-specific UPR systems involving the ATF6α family proteins play fundamental roles in the local homeostasis. OASIS (also known as cyclic AMP response element–binding protein 3 like protein 1; Creb3l1) or BBF2H7 (Creb3l2) are important for the bone formation [6], [7]. CREB4 (Creb3l4) is specifically expressed in testis and essential for the testicular spermatogenesis [8]. CREBH (Creb3l3) is also an ATF6-family member that is highly expressed in the liver and intestine [9]–[11]. To date, CREBH has been reported to mediate ER stress-dependent induction of acute-phase proteins in the liver, control of iron homeostasis by regulating the induction of hepcidin, and mediation of hepatic gluconeogenesis under a starvation condition [12]–[14].

The liver is a critical organ for detoxification of various drugs [15]. Recently, IRE1α is shown to be essential for protecting mice from hepatotoxicity of an analgesic drug acetoaminophen (APAP) [16]. XBP1-deficient mice displayed resistance to the APAP-induced fatal side effect due to feedback hepatic activation of IRE1α, leading to degradation of mRNA of cytochrome P450 genes such as CYP1A2 and CYP2E1, those of which play critical roles in oxidation of APAP to generate a major APAP metabolite that causes the fatal side effect. In addition, c-jun N-terminal kinase, which is activated in response to ER stress, has recently been identified as a critical determinant of the APAP-induced fatality [17], [18]. Thus, accumulating evidence suggests a potential link between hepatic ER stress and drug-induced fatality, however, the function of an ER stress protein CREBH that is remarkably expressed in the liver has not been studied so far in the context of drug-resistance or -susceptibility.

In this study, we show that CREBH-deficient mice are highly resistant to fatal shock induced by an antipyretic analgesic drug sulpyrine, but not by APAP. The hepatic mRNA expression of the CYP2B family members, which are important for the generation of sulpyrine metabolites, is severely reduced in CREBH-deficient mice. Moreover, ectopic expression of CREBH activates the CYP2B promoter, and sulpyrine treatment results in hepatic ER stress and nuclear translocation of CREBH. Furthermore, transient *in vivo* suppression of CREBH protects mice from sulpyrine-induced fatality. Thus, these results indicate that CREBH is critically involved in the sulpyrine-induced fatal shock by regulating hepatic expression of CYP2B family members.

## Results

### CREBH-deficiency confers mice on resistance to sulpyrine-induced shock

**Figure 1:**
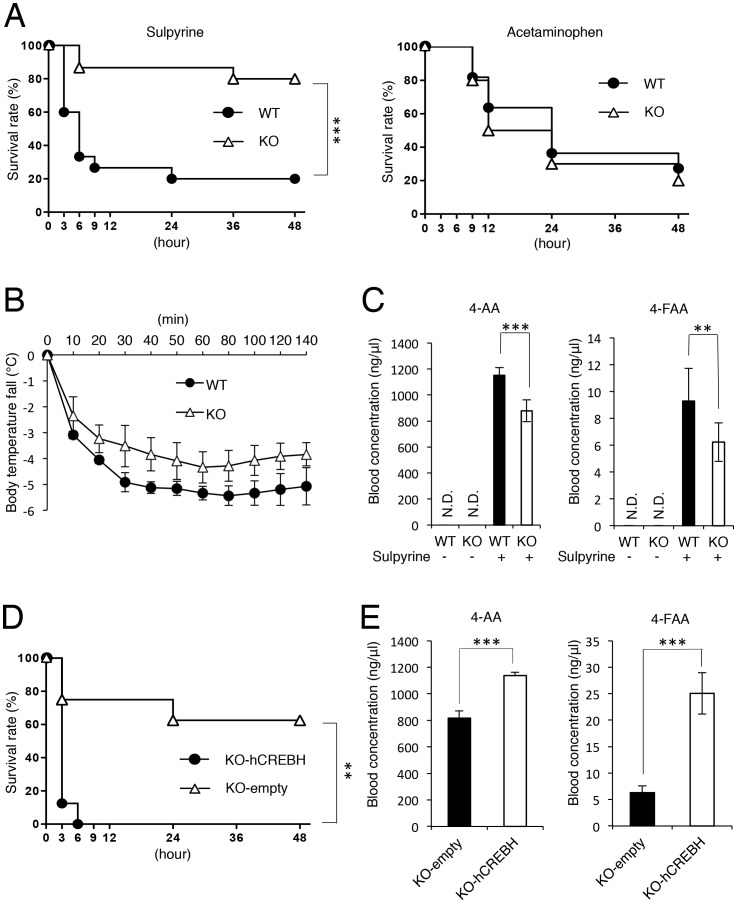
CREBH-deficient mice are resistant to fatal sulpyrine shock. (A) Wild-type (n = 15) and CREBH-deficient (n = 15) mice were intraperitoneally injected with 2.7 mg/g of sulpyrine (left). Wild-type (n = 11) and CREBH-deficient (n = 10) mice were intraperitoneally injected with 0.7 mg/g of acetaminophen (right). Survival rates were monitored for 48 hr, respectively. (B) Wild-type (n = 5) and CREBH-deficient (n = 5) mice were intraperitoneally injected with 2.7 mg/g of sulpyrine. Rectal temperature was measured at every 10 min for 140 min. (C) Wild-type (n = 10) and CREBH-deficient (n = 10) mice were intraperitoneally injected with 2.7 mg/g of sulpyrine. Sera were taken at 2 hr 40 min after sulpyrine injection. Serum concentrations of 4-AA and 4-FAA were measured by a HPLC assay. N.D., not detected. **, P<0.01; ***, P<0.001. (D) CREBH-deficient mice transfected with hCREBH expression vectors (n = 8) or empty vectors (n = 8) were intraperitoneally injected with 2.7 mg/g of sulpyrine. Survival rate was monitored for 48 hr. (E) CREBH-deficient mice transfected with hCREBH expression vectors (n = 3) or empty vectors (n = 3) were intraperitoneally injected with 2.7 mg/g of sulpyrine. Sera were taken at 2 hr 40 min after sulpyrine injection. Serum concentrations of 4-AA and 4-FAA were measured by a HPLC assay. ***, P<0.001. Data are pooled from two (A, D) independent experiments or representative of three (C) and two (B, E) independent experiments.

Because CREBH is remarkably expressed in the liver, we investigated the potential role of CREBH in detoxification by analysis of CREBH-deficient mice generated by gene targeting (Figure S1). We first analysed resistance to the pyrazolone-derived antipyretic analgesic drug sulpyrine and the non-pyrazolone antipyretic analgesic drug APAP in CREBH-deficient mice. Although high-dose acetaminophen administration resulted in comparable lethality, CREBH-deficient mice were resistant to sulpyrine (Figure 1A). Next, the antipyretic effect of sulpyrine was examined in wild-type and CREBH-deficient mice. The reduction of body temperature in sulpyrine-administrated mice was more gradual than that in wild-type mice (Figure 1B). Sulpyrine is non-enzymatically hydrolysed to 4-methylaminoantipyrine (4-MAA). Then, 4-MAA is further metabolized to 4-aminoantipyrine (4-AA) and 4-formylaminoantipyrine (4-FAA) in the liver (Figure S2) [19]. Therefore, sera were taken from sulpyrine-administrated mice just prior to the death of a number of wild-type mice, and the blood concentrations of 4-AA and 4-FAA were measured by a high-performance liquid chromatography (HPLC) assay (Figure S3). Compared with wild-type mice, sulpyrine-administrated CREBH-deficient mice exhibited lower concentrations of 4-AA and 4-FAA in their sera (Figure 1C). Given that 4-AA plays an important role in the sulpyrine-mediated antipyretic effect [20], [21], the mild antipyretic effect in CREBH-deficient mice is consistent with the lower concentrations of 4-AA and 4-FAA.

### CREBH increases serum level of the sulpyrine metabolite 4-AA

**Figure 2:**
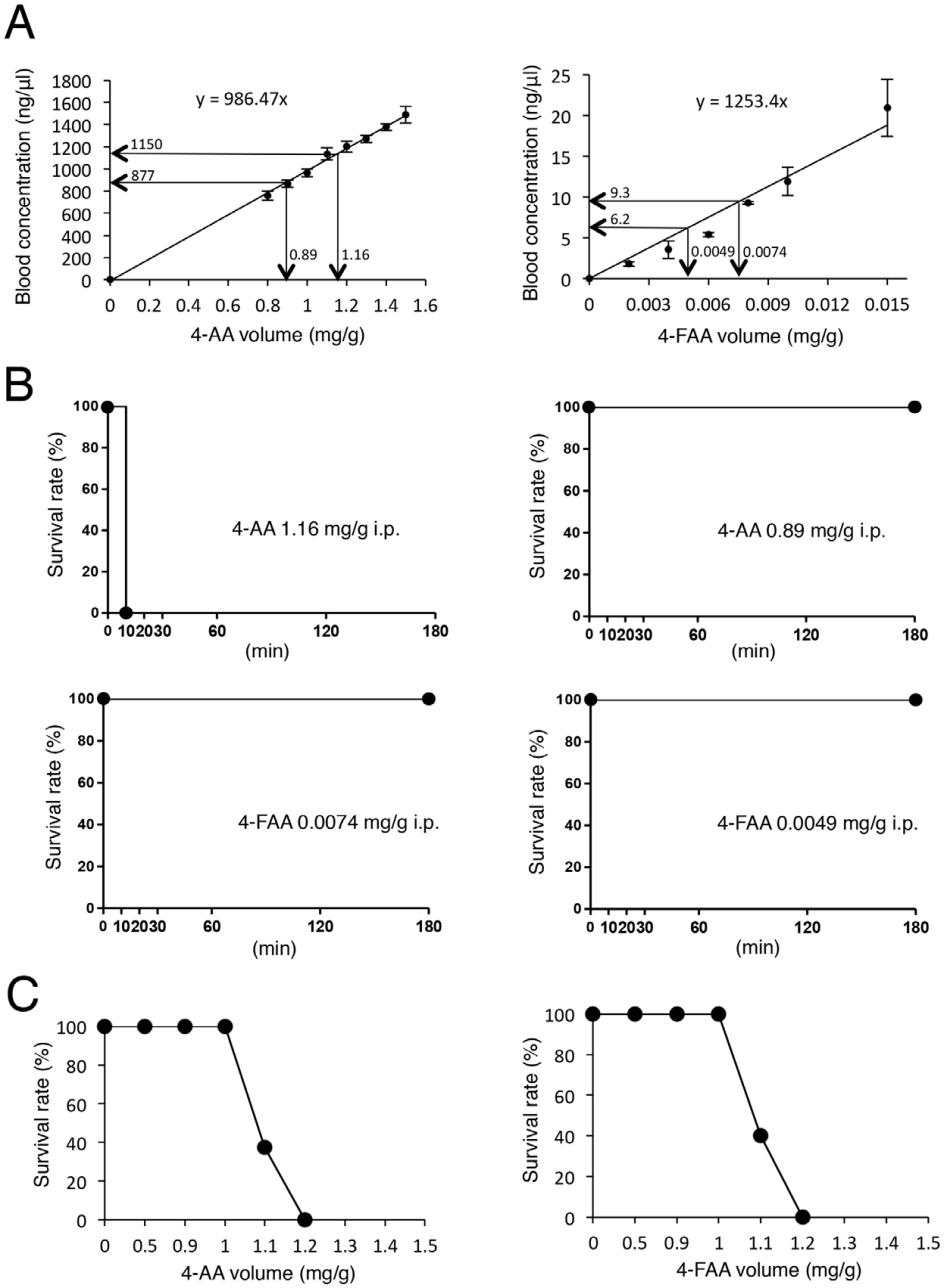
The blood concentration of 4-AA is involved in sulpyrine-induced shock. (A) Calibration curves for relationship between the administration volumes of 4-AA, 4-FAA and the concentrations of in the sera. (B) Wild-type mice (n = 5) were intraperitoneally injected with indicated volumes of 4-AA and 4-FAA. Survival rate was monitored for 180 min. (C) Wild-type mice (n = 5) were intraperitoneally injected with indicated volumes (0, 0.5, 0.9, 1.0, 1.2, 1.3, 1.4, and 1.5 mg/g) of 4-AA and 4-FAA. Survival rate was monitored for 180 min. Data are representative of two (A–C) independent experiments.

To determine which metabolite of sulpyrine was responsible for fatal sulpyrine shock, we directly administrated 4-AA or 4-FAA to wild-type mice. The relationship between the administration volume of both metabolites and their concentrations in the sera was analysed, and found to be directly proportional (Figure 2A). Next, we calculated the administration volume of 4-AA and 4-FAA by their concentrations in sera from wild-type and CREBH-deficient mice administrated with 2.7 mg/g sulpyrine (Figures 1C and 2A). In wild-type mice, blood concentrations of 1150 ng/µl 4-AA and 9.3 ng/µl 4-FAA corresponded to the administration volumes of 1.16 mg/g and 0.0074 mg/g, respectively. In addition, 0.89 mg/g 4-AA and 0.0049 mg/g 4-FAA were calculated to be administrated to CREBH-deficient mice (Figure 2A). Then, wild-type mice were separately challenged with the calculated administration volumes (Figure 2B). All mice administrated with both 4-FAA concentrations survived. In contrast, although all mice administrated with 0.89 mg/g 4-AA survived, those administrated with 1.16 mg/g 4-AA succumbed. Next, we examined the survival rate of mice administrated with various concentrations of both metabolites. 4-AA required almost similar concentrations to those in mice administrated with 2.7 mg/g sulpyrine. On the other hand, the lethal dose of 4-FAA was much higher than the calculated volume (Figure 2C). Together, these data suggest that the blood concentration of 4-AA, but not 4-FAA, is well correlated with sulpyrine-induced fatal shock. Furthermore, we administrated 1.1 mg/g 4-AA, in which 60% of mice did not survive, to wild-type mice, and compared the time-dependent blood concentrations of 4-AA in the sera between surviving and dead mice. Compared with surviving mice, dead mice showed higher blood concentrations of 4-AA in their sera. Considering the survival rate of both groups, almost 1000 ng/µl 4-AA in serum was the threshold between survival and death (Figure S4A). To further investigate the difference of sensitivity to 4-AA and 4-FAA between wild-type and CREBH-deficient mice, we administrated 1.1 mg/g 4-AA and 4-FAA to wild-type and CREBH-deficient mice. Consequently, no difference was found in the sensitivity to 4-AA and 4-FAA between wild-type and CREBH-deficient mice (Figure S4B). To confirm whether CREBH deficiency conferred resistance to sulpyrine-induced fatal shock, we transfected human CREBH expression vectors into CREBH-deficient mice *in vivo*, and tested whether reintroduction of CREBH expression restored sulpyrine-induced shock in CREBH-deficient mice. Hepatic expression of hCREBH mRNA and protein was confirmed by quantitative RT-PCR and immunohistochemical assays, respectively (Figure S5A and S5B). CREBH-deficient mice with introduced hCREBH showed a significantly lower survival rate than that of control mice (Figure 1D). Accordingly, blood concentrations of 4-AA and 4-FAA in hCREBH-introduced mice were markedly higher than those in control mice. In particular, blood concentrations of 4-AA exceeded the lethal concentration threshold (Figure 1E). Collectively, these data indicate that CREBH plays an important role in modulating the 4-AA concentration in serum and fatal shock induced by sulpyrine administration.

### CREBH-deficient mice are impaired in sulpyrine-induced expression of CYP2B family genes in the liver

**Figure 3:**
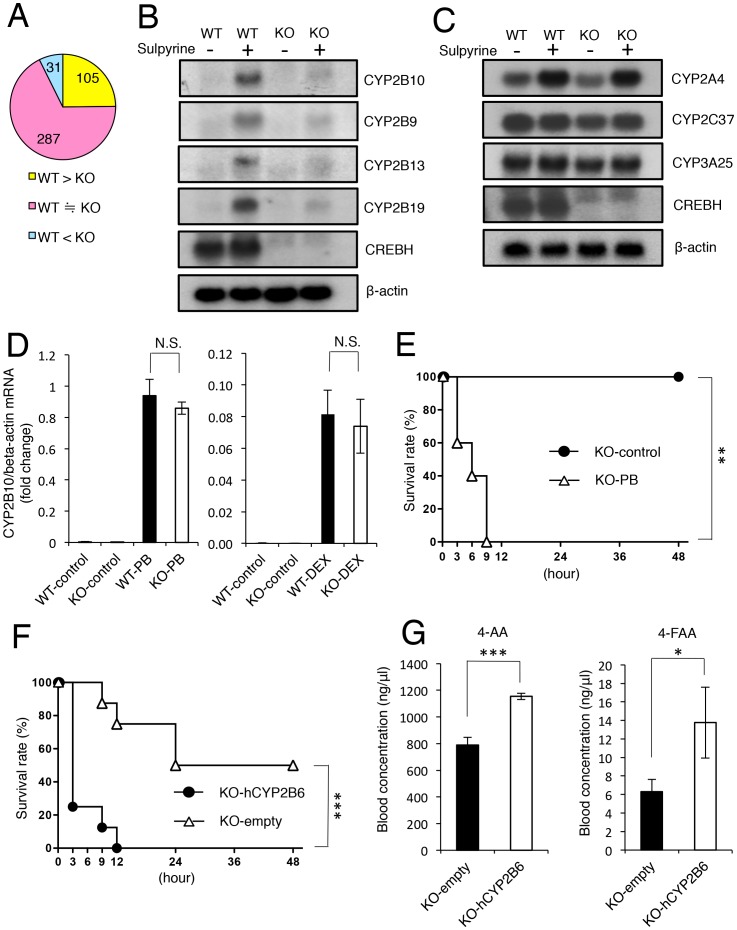
Sulpyrine-induced hepatic expression of CYP2B family genes is severely diminished in CREBH-deficient mice. (A) Summary of the microarray analysis. 423 sulpyrine-inducible genes were divided into down-regulated (yellow), similarly expressed (pink), and up-regulated (blue) groups, with the indicated amount of each. (B, C) Wild-type and CREBH-deficient mice were intraperitoneally injected with 2.7 mg/g of sulpyrine or vehicle alone (control). At 24 hr after injection, livers were taken from these mice. Total RNA was extracted and subjected to northern blot analysis for expression of the indicated probes. The same membrane was rehybridized with a β-actin probe. (D) Wild-type mice and CREBH-deficient mice were intraperitoneally injected with 100 mg/kg phenobarbital (n = 3) or vehicle alone (control) (n = 3) every 24 hr for 3 days (left), or were intraperitoneally injected with 50 mg/kg dexamethasone (n = 7) or vehicle alone (control) (n = 3) (right). At 24 hr after the last injection, livers were taken from these mice. Gene expression of CYP2B10 was analysed by a quantitative RT-PCR assay. N.S., not significant. (E) CREBH-deficient mice were pre-treated with 100 mg/kg PB (n = 5) or vehicle alone (control) (n = 5) every 24 hr for 3 days. At 24 hr after the last injection, the mice were intraperitoneally injected with 1.1 mg/g of sulpyrine. Survival rate was monitored for 48 hr. (F) CREBH-deficient mice transfected with hCYP2B6 expression vectors (n = 8) or empty vectors (n = 8) were intraperitoneally injected with 2.7 mg/g of sulpyrine. Survival rate was monitored for 48 hr. (G) CREBH-deficient mice transfected with hCYP2B6 expression vectors (n = 3) or empty vectors (n = 3) were intraperitoneally injected with 2.7 mg/g of sulpyrine. Sera were taken at 2 hr 40 min after sulpyrine injection. Serum concentrations of 4-AA and 4-FAA were measured by a HPLC assay. *, P<0.05; ***, P<0.001. Data are pooled from two (E) independent experiments or representative of two (A, B, C, D, F) independent experiments.

Because CREBH is highly expressed in the liver, we performed microarray analysis of hepatic samples from wild-type and CREBH-deficient mice to compare gene expression profiles in response to sulpyrine. Out of 22690 transcripts, we first defined genes up-regulated by more than 2.5-fold after sulpyrine administration to wild-type mice as “sulpyrine-inducible genes” and identified 423 such genes. We next compared sulpyrine-inducible genes in wild-type and CREBH-deficient mice after sulpyrine administration, and found that 105, 287 and 31 genes were down-regulated, similarly expressed and up-regulated in CREBH-deficient mice, respectively (Figure 3A and Table S1). Microarray analysis also revealed that among the down-regulated genes, expression of CYP2B family genes was severely impaired in CREBH-deficient mice. CYP2B family genes play a role in sulpyrine metabolism [22]–[24]. Therefore, we tested whether CYP2B family genes in the liver were induced in sulpyrine-administered mice by northern blot analysis. Sulpyrine administration to wild-type mice resulted in strong mRNA expression of CYP2B family genes. On the other hand, sulpyrine-induced expression was much lower in livers from sulpyrine-administrated CREBH-deficient mice (Figure 3B), whereas no difference was found in the expression of other cytochrome P450 family genes, such as CYP2A4, CYP2C37 and CYP3A25, which are not involved in sulpyrine metabolism (Figure 3C). These results suggest that sulpyrine shock in CREBH-deficient mice might be suppressed due to the low expression of CYP2B family genes. CYP2B gene has been shown to be induced by phenobarbital or dexamethasone, which activates constitutive androstane receptor (CAR) or pregnane X receptor (PXR) respectively [25]–[29]. We found robust induction of CYP2B family genes in the livers from CREBH-deficient mice treated with phenobarbital or dexamethasone by quantitative RT-PCR assays (Figure 3D), suggesting that CREBH is not involved in CAR-mediated or PXR-mediated induction of CYP2B family genes. Furthermore, CREBH-deficient mice pre-treated with phenobarbital were sensitive to sulpyrine (Figure 3E), indicating that hepatic expression of CYP2B genes induced by phenobarbital may confer sensitivity to sulpyrine in CREBH-deficient mice. To test this hypothesis directly, we administered a human CYP2B6 expression vector to CREBH-deficient mice *in vivo*. Quantitative RT-PCR and immunohistochemical assays confirmed hepatic expression of hCYP2B6 mRNA and protein, respectively (Figure S6A and S6B). CREBH-deficient mice with hCYP2B6 expression were more sensitive to sulpyrine than control mice (Figure 3F). Moreover, blood concentrations of 4-AA and 4-FAA in hCYP2B6-expressing mice were higher than those in control mice (Figure 3G). Together, these results demonstrated that the mechanism by which CREBH-deficient mice exhibit resistance to sulpyrine is due to the lack of sulpyrine-induced expression of CYP2B family genes in their livers.

### CREBH activates the CYP2B10 promoter

**Figure 4:**
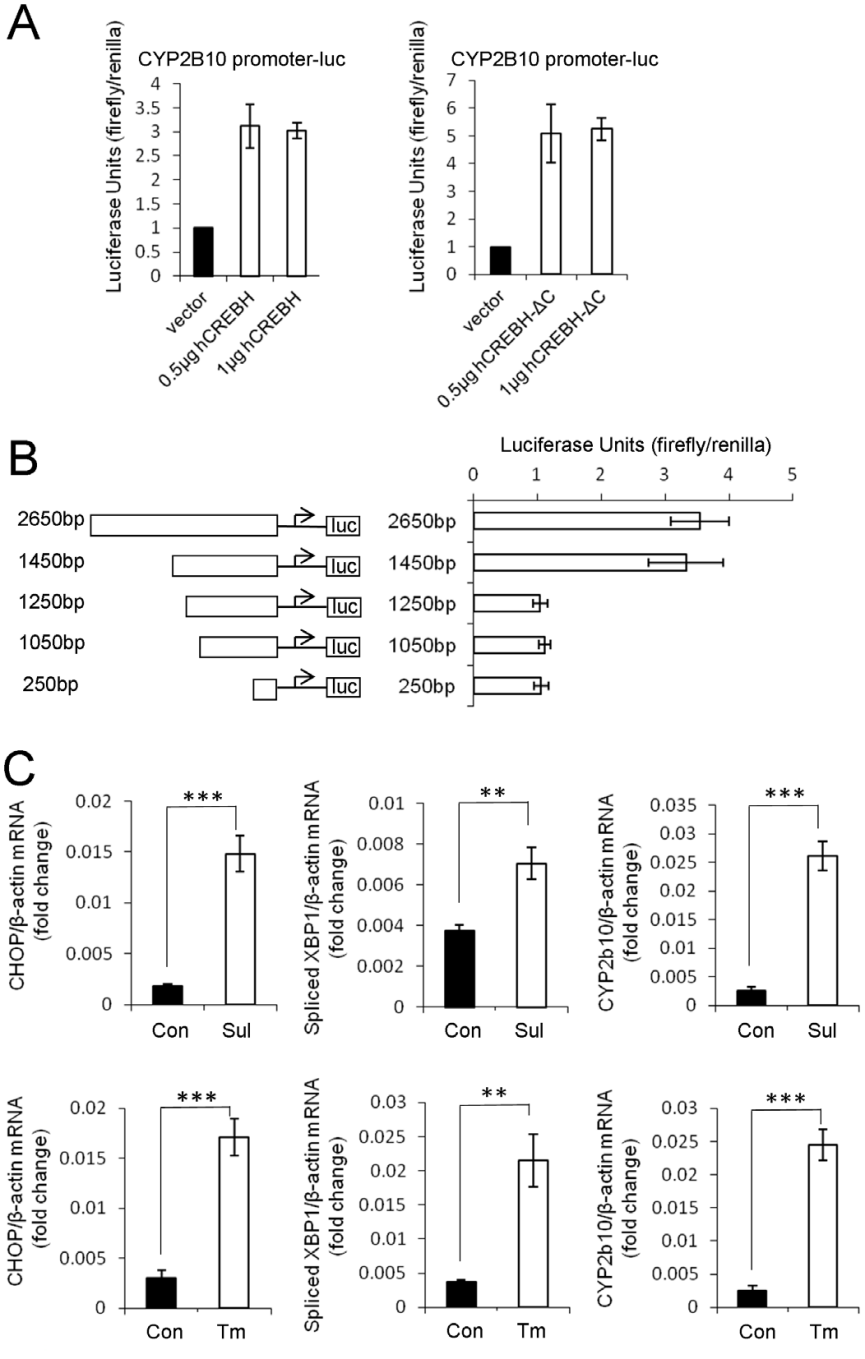
Sulpyrine treatment causes ER stress. (A) Huh7 cells were transiently transfected with luciferase reporter plasmids containing the CYP2B10 promoter together with control, hCREBH-F, or hCREBH-ΔC expression vector. Relative luciferase activities were shown as fold increases over the background levels shown by lysates prepared from control vector-transfected cells. Error bars are means ± S.D. of triplicates. (B) Huh7 cells were transiently transfected with luciferase reporter plasmids containing the indicated length CYP2B10 promoter together with control or hCREBH-F expression vector. Relative luciferase activities were shown as fold increases over the background levels shown by lysates prepared from control vector-transfected cells. Error bars are means ± S.D. of triplicates. (C) Wild-type mice were intraperitoneally injected with 2.7 mg/g of sulpyrine (n = 3) or 200 µg/kg tunicamycin (n = 3) or vehicle alone (control) (n = 3). At 2 hr 40 min after injection, hepatocytes were taken from these mice by a perfusion apparatus. Gene expression of CHOP, spliced XBP1, and CYP2B10 was measured by a quantitative RT-PCR assay. **, P<0.01; ***, P<0.001. Data are representative of three (A, B) and two (C) independent experiments.

To examine whether CREBH controlled the expression of CYP2B genes, we generated a luciferase reporter plasmid harbouring the CYP2B10 promoter, and introduced the reporter plasmid into Huh7 cells together with CREBH expression vectors to measure the promoter activity by a luciferase assay. Overexpression of full-length CREBH activated the CYP2B10 promoter. In addition, promoter activation by ectopic expression of CREBH lacking the C-terminus (CREBH-ΔC), a constitutively active form of CREBH, was more enhanced than that by full-length CREBH (Figure 4A), indicating that CREBH positively regulates the expression of the CYP2B10 gene. Subsequently, to determine a CREBH responsive region in the CYP2B10 promoter, reporter plasmids harbouring various lengths of the CYP2B10 promoter were constructed, and the promoter activities were measured by a luciferase assay. Reporters containing 2650 or 1450 bp were both activated. In contrast, reporters containing 1250 bp or less were not activated, suggesting that the CREBH responsive region may be located between −1450 and −1250 bp in the CYP2B10 promoter (Figure 4B). In addition to CREBH, CAR positively regulates CYP2B10 gene expression. The CAR-responsive element, called the Nr1 region, is located at −2350 bp (Figure S7A) [30]. To analyse whether CAR was involved in CREBH-dependent activation of the CYP2B10 promoter, we compared the luciferase activities of reporters containing and lacking Nr1 in the presence of CAR or CREBH. Activation of the Nr1-containing reporter in the prescience of CAR was stronger than that lacking Nr1. Conversely, in the presence of CREBH, both reporters were similarly activated (Figure S7B). Moreover, we isolated hepatocytes from wild-type and CREBH-deficient mice by perfusion, introduced CYP2B10 promoter reporter plasmids containing Nr1 into the hepatocytes together with CAR expression vectors, and then performed a luciferase assay. CAR-induced activation of the CYP2B10 promoter was comparable between wild-type and CREBH-deficient hepatocytes (Figure S7C). These results indicated that CREBH and CAR may independently regulate hepatic expression of the CYP2B10 gene, and may be consistent with the results of phenobarbital-treated CREBH-deficient mice, in which the CYP2B gene was induced in a CAR-dependent manner, thereby conferring sensitivity to sulpyrine (Figure 3D and 3E).

### CREBH is activated by sulpyrine-induced ER stress

**Figure 5:**
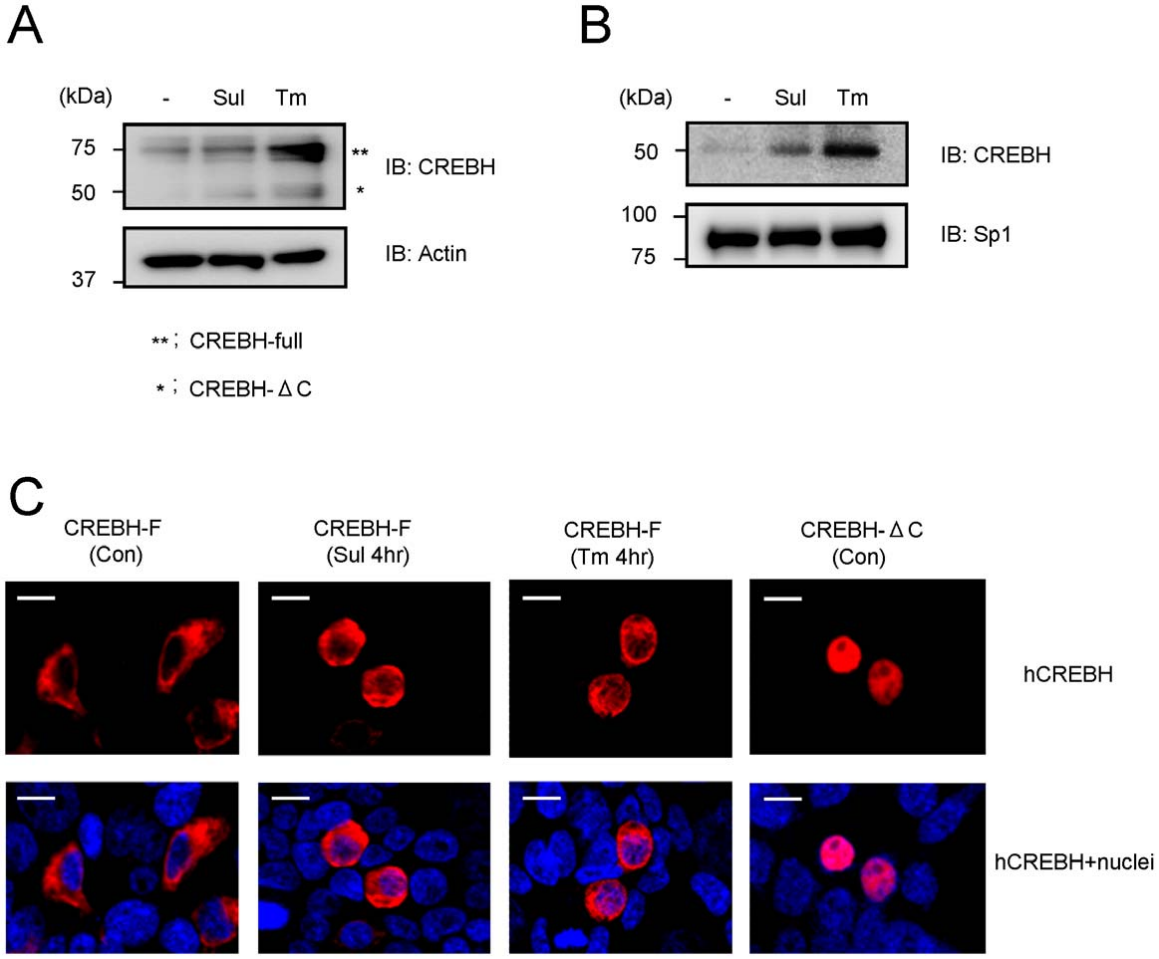
Cleavage of CREBH and its translocation into nucleus were induced by sulpyrine. (A) 293T cells were transiently transfected with 2 µg HA-tagged hCREBH-F expression vectors, and they were cultured with sulpyrine (2 mM) or tunicamycin (5 µg/ml) for 6 hr. Whole-cell lysates were prepared and the size of CREBH was analysed by western blot analysis using anti-CREBH monoclonal (upper panel) and anti-actin antibodies (lower panel). **, CREBH-full ; *, CREBH-ΔC. (B) 293T cells were transiently transfected with 2 µg HA-tagged hCREBH-F expression vectors, and they were cultured with sulpyrine (2 mM) or tunicamycin (5 µg/ml) for 6 hr. The cell lysates of nuclear fractions were prepared. The nuclear translocation of CREBH was analyzed by western blot analysis using anti-CREBH monoclonal (upper panel) and anti-Sp1 monoclonal antibodies (lower panel). (C) 293T cells were transiently transfected with HA-tagged hCREBH-F expression vectors and HA-tagged hCREBH-ΔC expression vectors, and they were cultured with mock, sulpyrine (2 mM) and tunicamycin (5 µg/ml) for 4 hr. Cells were stained with anti-HA mouse antibody, then stained with Alexa Fluor 594-conjugated anti-mouse IgG (red) together with DAPI (blue). Stained cells were analysed using a confocal microscope. Bars, 10 µm. Data are representative of three (A) and two (B,C) independent experiments.

Previous studies have shown that CREBH is activated in an ER stress-dependent manner, and mediates the expression of genes associated with the unfolded protein response [9], [12], [31]. To clarify whether hepatic ER stress was induced by sulpyrine administration, we isolated hepatocytes from wild-type sulpyrine- and tunicamycin-administrated mice, and then measured the expression of CCAAT/enhancer binding protein homologous protein (CHOP) mRNA and the spliced form of X-box binding protein 1 (XBP1), indicator of ER stress, by a quantitative RT-PCR assay [12]. Sulpyrine administration similarly induced the expression of CHOP mRNA and splicing of XBP1 mRNA in response to tunicamycin (Figure 4C). To test whether CREBH was cleaved and its N-terminus was translocated into the nucleus upon stimulation with sulpyrine, we introduced CREBH expression vectors into 293T cells, and then analysed the cleavage and localization of CREBH by western blot and immunofluorescence assay, respectively. Notably, sulpyrine treatment caused the cleavage of CREBH (Figure 5A) and nuclear translocation of CREBH (Figure 5B). The extent of sulpyrine-induced nuclear localization of CREBH was similar to that by tunicamycin, while CREBH-ΔC was steadily localized in the nucleus without stimulation (Figure 5C). Taken together, these results indicate that sulpyrine administration possibly induces ER stress in the liver and results in activation of CREBH, leading to expression of the CYP2B10 gene.

### The CREBH-knockdown mice exhibit resistance to fatal sulpyrine shock

**Figure 6:**
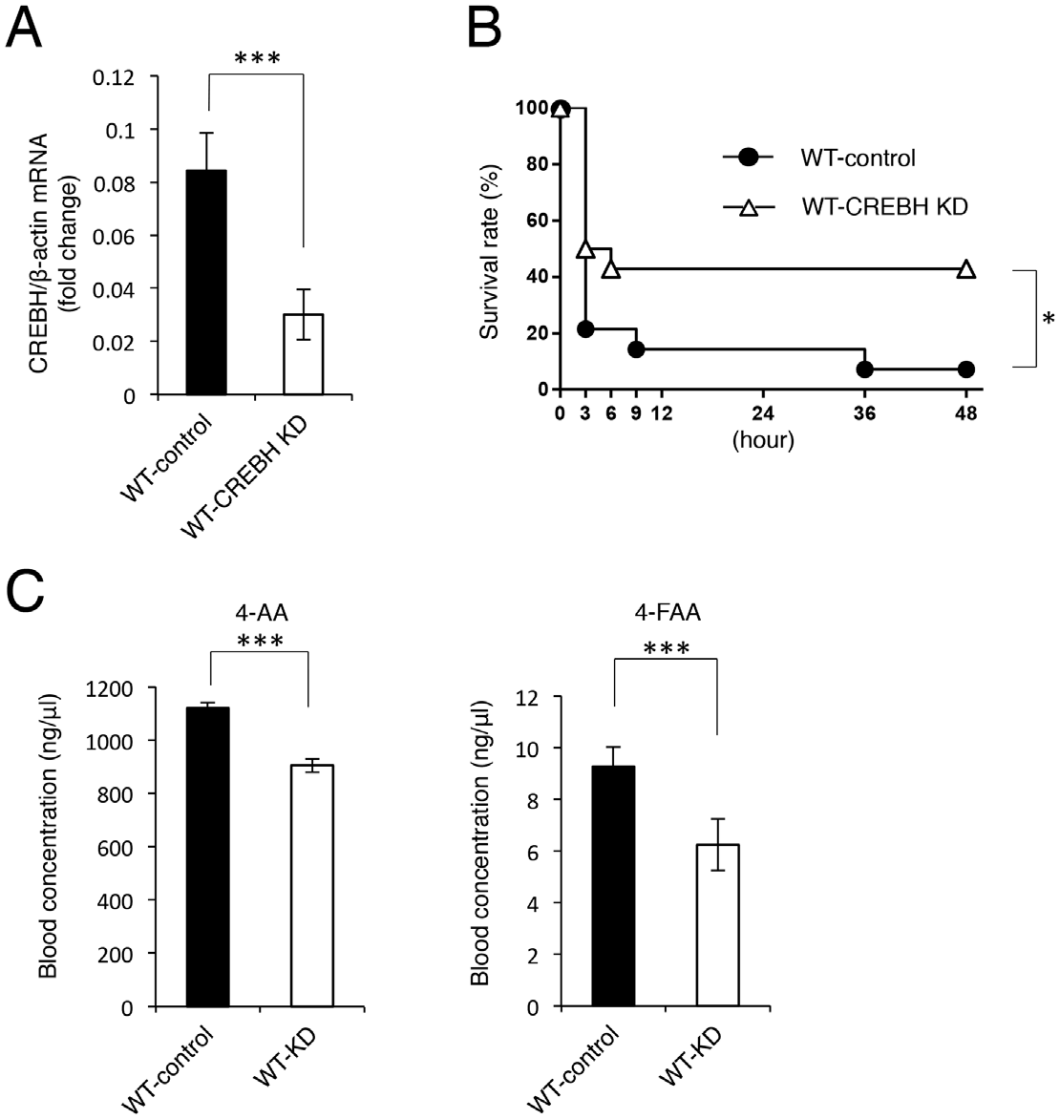
RNAi-mediated CREBH-knockdown mice are resistant to the sulpyrine shock. (A) Livers of wild-type mice transfected with RNAi vectors for CREBH (n = 4) or negative control vectors (n = 4) were taken at 24 hr after transfection. Gene expression of CREBH was analysed by a quantitative RT-PCR assay. ***, P<0.001. (B, C) Wild-type mice transfected with RNAi vectors for CREBH (n = 14) or negative control vectors (n = 14) were intraperitoneally injected with 2.7 mg/g of sulpyrine at 24 hr after transfection. Survival rate was monitored for 48 hr. *, P<0.05. Sera were taken at 2 hr 40 min after sulpyrine injection. Serum concentrations of 4-AA and 4-FAA were measured by a HPLC assay. ***, P<0.001. Data are representative of two (A, C) independent experiments or pooled from three (B) independent experiments.

Despite the strong antipyretic effect, fatal shock induced by sulpyrine is so clinically problematic that use of this drug is restricted and even prohibited in several countries [32]. Therefore, fatality should be precluded in advance for the safe usage of sulpyrine. We attempted to prevent sulpyrine shock by transient reduction of CREBH in wild-type mice. First, we selected an efficient RNAi vector against CREBH *in vitro* (Figure S8), transfected the RNAi vector into wild-type mice *in vivo*, and confirmed the efficiency of *in vivo* knockdown in the liver by a quantitative RT-PCR assay (Figure 6A). Next, we administrated sulpyrine to CREBH-knockdown mice, and then measured the survival rate and blood concentrations of 4-AA and 4-FAA. CREBH-knockdown mice exhibited resistance to fatal sulpyrine shock, compared with that of control mice (Figure 6B). In addition, blood concentrations of 4-AA and 4-FAA in CREBH-knockdown mice were lower than those in control mice (Figure 6C). Thus, these results suggest that RNAi-mediated suppression of CREBH can alleviate fatal sulpyrine shock.

## Discussion

It has been almost a century since sulpyrine became a widely-used therapeutic drug. Despite the severe side effects, sulpyrine is generally used both in the adults and children as a first-line antipyretic analgesic in some developed countries as well as a number of developing countries [32]–[35]. Severe shock is a fatal side effect of pyrazolone derivatives including sulpyrine. The peculiar symptoms such as loss of consciousness, coma, and convulsions are associated with the shock, eventually leading to a lethal condition or death in some patients [36], [37]. In fact, severe convulsions were also observed in sulpyrine-administrated mice in our study (data not shown). Therefore, sufficient caution should be exercised in sulpyrine medication. However, pathogenic mechanism of sulpyrine-induced shock is poorly understood. In this study, we identify that 4-AA, one of the sulpyrine metabolites, is the causative substance of sulpyrine-induced shock. In addition to sulpyrine, another pyrazolone derivative, aminopyrine is metabolized to 4-AA [38], and cause anaphylactic shock as a side effect [39], suggesting that 4-AA may be the causative substance of the shock.

In this study, we demonstrate that CREBH mediates sulpyrine-induced fatal shock. Upon ER stress, CREBH is previously reported to translocate to the Golgi apparatus, and cleaved by S1P and S2P as well as SREBPs. The cleaved cytosolic lesion of the N-terminal CREBH traffics to the nucleus [12]. We show that CREBH was activated by sulpyrine-induced ER stress, and trafficked to the nucleus to activate transcriptional induction of CYP2B family genes in the liver. CYP2B induced by CREBH increased the serum concentration of 4-AA, which has the antipyretic effect. Furthermore, CREBH-deficient mice with ectopic expression of hCREBH or CYP2B6 became sensitive to sulpyrine, suggesting that CREBH or CYP2B was a critical trigger for the sulpyrine-induced fatal shock. CAR and PXR are known as the transcriptional activators of CYP2B family genes. Although, we originally hypothesized that CREBH was involved in the induction of CYP2B family genes collaborated with CAR or PXR, our data suggested that CREBH induced transcription of CYP2B family genes independently of either CAR or PXR.

APAP is a widely used antipyretic analgesic drug. It has been recently reported that hepatic XBP1-deficient mice were resistant to APAP-induced hepatic dysfunction due to low expression levels of mRNAs of CYP1A2 and CYP2E1, both of which are metabolic enzymes for APAP to produce NAPQI by hydroxylation of acetaminophen [16], [40]–[42]. NAPQI is normally detoxified by glutathione conjugation. However, NAPQI is accumulated in the liver by the mass administration of APAP and the hepatic dysfunction, it binds to biopolymer which induces hepatic necrosis [43]. Although subclasses of CYP enzymes, which expression were reduced in the mutant mice were different (i.e. CYP2B or CYP1A2/CYP2E1 for CREBH or XBP1, respectively), mice deficient in ER stress related transcription factors showed resistance to the fatal side effect of antipyretic analgesic drugs because of the low CYP expression. However, the mechanism of the reduced CYP expression by the deficiency of each transcription factor is different. The XBP1 deficiency leads to upregulation of the ribonuclease IRE1α, which degrades mRNA for CYP1A2/CYP2E1, suggesting the indirect reduction of CYP1A2/CYP2E1 by the XBP1-deficiency [16]. On the other hand, CREBH directly induces CYP2B expression upon sulpyrine stimulation. The difference might be caused by the property or injected dose of these drugs. Furthermore, tunicamycin, which is known to activate CREBH, upregulated CYP2B10 strongly in the liver (Figure 4C). Thus, other hepatic ER stress inducible drugs might be able to induce CYP2B family genes via CREBH activation.

Recently, some NSAIDs have been reported to induce ER stress. Indomethacin treatment induces ER stress in murine podocytes [44], and CHOP expression in gastric mucosal cells of guinea pig, resulting in apoptosis [45]. The CHOP-mediated apoptosis is also stimulated by other NSAIDs such as celecoxib and sulindac sulfide, in human gastric cells and colon epithelial cancer cells, respectively [46] [47]. The gastric ulcer is the representative side effect in NSAIDs [48]. On the other hand, the incidence rate of cancer has been shown to lower by a long-term NSAIDs medication [49], [50]. Although these molecular mechanisms remain unclear, the involvement of NSAIDs-induced ER stress in these phenomena is suggested [51], [52]. Treatment of some NSAIDs such as indomethacin, diclofenac, etodolac, ibuorofen, celecoxib, and ketoprofen increase intracellular Ca^2+^ levels, and stimulate Ca^2+^ influx across the cytoplasmic membrane, leading to the increment of membrane permeability, mitochondrial dysfunction, and apoptosis [53]. Whether Ca^2+^ is also involved in sulpyrine-induced ER stress should be examined in the future.

In this study, we demonstrate that CREBH-dependent expression of CYP2B in the liver is one of the molecular mechanisms of fatal shock induced by sulpyrine in a mouse model. However, whether this mechanism also applies to humans remains unclear and it would be of interest to test whether the hepatic expression level of CREBH (and CYP2B) is relevant to sulpyrine-induced shock in humans. Furthermore, given that transient knockdown of CREBH prior to sulpyrine administration mitigates the shock in mice, therapeutic application of a molecule that inhibits CREBH action might be beneficial for patients that are sensitive to sulpyrine.

## Materials and Methods

### Generation of CREBH-deficient mice

The CREBH gene was isolated from genomic DNA extracted from ES cells (V6.5) by PCR using TaKaRa LA Taq™ (TaKaRa) [54]. The targeting vector was constructed by replacing a fragment encoding the exons of *CREBH* with a neomycin-resistance gene cassette (neo), and a herpes simplex virus thymidine kinase driven by PGK promoter was inserted into the genomic fragment for negative selection. A genomic DNA containing the murine *CREBH* gene was isolated from PCR amplification by using primer CREBHKO_LA_F and CREBHKO_LA_R, to generate a 5.0 kb long fragment, and CREBHKO_SA_F and CREBHKO_SA_R, to generate a 1.0 kb short fragment. The sequences of primers are listed in Table S2. After the targeting vector was transfected into ES cells, G418 and gancyclovir doubly resistant colonies were selected and screened by PCR and southern blotting using the probe indicated in Fig S1A. Homologous recombinants were micro-injected into C57BL/6 female mice, and heterozygous F1 progenies were intercrossed in order to obtain CREBH-deficient mice. CREBH-deficient mice and their wild-type littermates from these intercrosses were used for experiments. Male mice (8–11 weeks old; approximately 25 g weight) were used in the present study. All animal experiments were conducted with approval of the Animal Research Committee of the Graduate School of Medicine at Osaka University.

### Reagents

Sulpyrine Monohydrate, Phenobarbital Sodium were purchased from Wako. Dexamethasone was purchased from SIGMA. 4-Aminoantipyrine was purchased from nacalai tesque. 4-Formylaminoantipyrine was purchased from Aldorich Chemistry. Tunicamycin was purchased from Enzo Life Science. Anti-HA HRP, anti-actin HRP, anti-CREBH goat polyclonal immunoglobulin G, anti-goat immunoglobulin G HRP, anti-Sp1 rabbit polyclonal immunoglobulin G were purchased from Santa cruz. Alexa Fluor 488-conjugated anti-mouse immunoglobulin G, Alexa Fluor 594-conjugated anti-mouse immunoglobulin G, anti-rabbit immunoglobulin G HRP were purchased from Invitrogen. 4, 6-diamidino-2-phenylindole (DAPI) was purchased from Wako.

### High performance liquid chromatography

HPLC equipment, consisting of Nanospace SI-2/3201 pump (SHISEIDO), Nanospace SI-2/3023 autosampler (SHISEIDO), Nanospace SI-2/3010 degasser (SHISEIDO), and Accela PDA Detector (Thermo Scientific ) was used. A detector was operated at 254 nm wavelength. Separation was achieved with an μBondapak™ C18 10 µm 125 A 3.9×150 mm column (Waters). The following eluent were used: 8% methanol in 0.01 M sodium acetate, adjusted to pH 3.0 with concentrated hydrochloric acid [55]. The column was maintained at room temperature and the flow-rate was 1000 µl/min. Serum samples were directly injected into the system after simple filtration with a membrane filter. Aliquots of 4-AA and 4-FAA stock solution were diluted in the eluent and analyzed with the HPLC to obtain a standard curve. We compared the difference of the area under the curve of the sera with stock solution.

### RNA interference

293T cells were cotransfected with 2 µg or 1 µg RNAi vectors targeting mouse CREBH in BLOCK-iT Pol II miR RNAi Expression Vector Kit (Invitrogen) and HA-tagged mCREBH expression vector. 24 hr after transfection, whole-cell lysates were prepared and subjected to Western blot analysis.

### *In vivo* transfections

Twenty micrograms of expression plasmids for hCREBH or hCYP2B6, or RNAi vectors for mCREBH were mixed with 30 µl Lipofectamine 2000 reagent (Invitrogen) in 170 µl of Phosphate buffered saline (PBS), and the complexes were injected intravenously via orbital sinus [56].

### Expression plasmids

Human CREBH cDNA was amplified using primers hCREBH_full_R for Full, hCREBH_ΔC_R for ΔC, and the common primer hCREBH_common_F using human liver cDNA as the template and then ligated into the *EcoRI* and *NotI* sites of a pcDNA vector for the N-terminal HA-tagged proteins (Invitrogen). Human CYP2B6 cDNA was amplified using primers CYP2B6_F and CYP2B6_R using human liver cDNA as the template and then ligated into the *BamHI* and *NotI* sites of a pcDNA vector for the N-terminal HA-tagged proteins. Human CAR cDNA was amplified using primers CAR_F and CAR_R using human liver cDNA as the template and then ligated into the *EcoRI* and *NotI* sites of a pcDNA vector for the N-terminal HA-tagged proteins. Murine CREBH cDNA was amplified using primers mCREBH_F and mCREBH_R using murine liver cDNA as the template and then ligated into the and *BglII* and *NotI* sites of a pcDNA vector for the N-terminal HA-tagged proteins. The sequences of primers are listed in Table S2.

### Real-time RT-PCR

Total RNA of livers and hepatocyte were isolated with TRIzol reagent (Invitrogen), cDNA was generated using 5 µg of RNA and Verso cDNA Kit (Thermo Scientific). Real-time RT-PCR was performed on an ABI 7300 real-time PCR system (Applied Biosystems) using GoTaq qPCR Master Mix (Promega). All data were normalized to the β-actin expression, and the fold difference relative to the β-actin was shown. Amplification conditions were: 50°C (2 min), 95°C (10 min), 40 cycles of 95°C (15 s), and 60°C (60 s). Primers of hCREBH, mCREBH, CYP2B6, CYP2B10, CHOP, spliced XBP1 and β-actin were purchased from Invitrogen. The sequences of primers are listed in Table S2.

### Immunohistochemical assay

Wild-type mice were transfected with HA-tagged pcDNA-CREBH or CYP2B6 expression vectors *in vivo*. At 24 hr after transfection, livers were taken and fixed with 4% paraformaldehyde (Wako) and cryoprotected in 10% sucrose for 10 min and 20% sucrose for 10 min, and embedded in OCT compound (SAKURA). 8 µm in thickness of liver cryostat sections were fixed in acetone, and washed with PBS. Sections were incubated with anti-HA (1∶100) in Tris-HCl buffer saline (TBS) containing 1% bovine serum albumin, then incubated with Alexa Fluor 488-conjugated anti-mouse immunoglobulin G (1∶100). To stain the nucleus, sections were cultured with DAPI (1∶10000). The stained sections were mounted with Mountant, PermaFluor (Thermo SCIENTIFIC) and analyzed using a fluorescence microscope (FV1000-D IX81; Olympus).

### Microarray analysis

Total RNA of livers were isolated with TRIzol reagent (Invitrogen), RNA quality was assessed with an Agilent Bioanalyser 2100 and only RNA with minimal degradation and distinct 18S and 28S rRNA bands were used for analysis. Fragmented and biotin-labeled cDNA was synthesized from 100 ng purified mRNA with GeneChip 3′IVT Express Kit Assay (Affymetrix). The cDNA was hybridized to Mouse Genome 430A 2.0 Array (Affymetrix). Hybridized chips were stained and washed and were scanned with GeneChip Scanner 3000 (Affymetrix). Genespring software (Silicon Genetics) was used for data analysis.

### Northern blot analysis

Total hepatic RNA was extracted using the TRIzol reagent (Invitrogen). Fifteen µg of total RNA was electrophoresed, transferred to a nylon membrane, and hybridized with cDNA probes as described previously [57]. cDNA probes specific for CREBH, β-actin, CYP2B10, CYP2B9, CYP2B13, CYP2B19, CYP2A4, CYP2C37, and CYP3A25 were obtained by PCR with a set of specific primers from a mouse liver cDNA. The sequences of primers are listed in Table S2.

### Luciferase reporter assay

The Luciferase reporter plasmids were transiently cotransfected into Huh7 cells with the control Renilla luciferase expression vectors using Lipofectamine 2000 reagent (Invitrogen). Luciferase activities of total cell lysates were measured using the Dual-Luciferase Reporter Assay System (Promega). The CYP2B10 promoter was amplified using primers CYP2B10 promoter_F for 1450 bp, CYP2B10 promoter_F for 1250 bp, CYP2B10 promoter_F for 1050 bp, CYP2B10 promoter_F for 250 bp, and CYP2B10 promoter_common_R using murine genomic DNA as the template. Luciferase reporter was generated by *MluI* fragment of the insertion the CYP2B10 promoter into the *MluI* site of pGL3 luciferase reporter. Luciferase reporter harboring CYP2B10 promoter for 2650 bp was generated by inserting the *MluI* fragment of the CYP2B10 promoter for 2650 bp into the *MluI* site of luciferase reporter harboring CYP2B10 promoter for 1450 bp. The CYP2B10 promoter for 2650 bp was amplified using primers CYP2B10 promoter_F for 2650 bp and CYP2B10 promoter_R for 2650 bp using murine genomic DNA as the template. The sequences of primers are listed in Table S2.

### Preparation of primary mouse hepatocytes

Mice were anaesthetized, and subsequently the abdomen was opened to expose the liver. The perfusion needle (26 G TERUMO) was inserted into the portal vein, and the perfusion was started with 50 ml 37°C SC-1 solution consisting of 8000 mg/l NaCl, 400 mg/l KCl, 88.7 mg/l NaH2PO4·H2O, 120.45 mg/l Na2HPO4, 2380 mg/l HEPES, 350 mg/l NaHCO3, 190 mg/l EGTA, and 900 mg/l glucose (pH 7.25). When the perfusion was started immediately after cutting the ventral aorta. After all of SC-1 solution was flowed, the perfusion was continued to 100 ml 37°C SC-2 solution consisting of 8000 mg/l NaCl, 400 mg/l KCl, 88.7 mg/l NaH2PO4·H2O, 120.45 mg/l Na2HPO4, 2380 mg/l HEPES, 350 mg/l NaHCO3, and 560 mg/l CaCl2·2H2O (pH 7.25) added 0.2 µg/ml collagenase D from *Clostridium histolyticum* (Roche) and 0.02 µg/ml trypsin inhibitor from chicken egg white (SIGMA). After all of SC-2 buffer was flowed, steeped the liver to SC-2 buffer, and breached a hepatic capsule, and shaken gently on ice. The liver was crumbled gently with an autopipetter, and filtered with a gauze. The hepatocytes were separated by low speed centrifugation (400 r.p.m.), and the obtained pellets were washed with ice-cold SC-2 buffer to purify the cells [58].

### Immunofluorescence analysis

293T cells were transfected with 2 µg HA-tagged hCREBH expression vectors by Lipofectamine 2000 reagent (Invitrogen). After 24 hr, the cells were stimulated with 2 mM sulpyrine or 5 µg/ml Tunicamycin for 4 hr, and washed with PBS, and then fixed with 4% paraformaldehyde (Wako) for 10 min at room temperature. After 15 min permeabilization with 0.2% Triton X-100, cells were washed with PBS, and blocked with Blocking One (nacalai tesque) for 10 min, and incubated with anti-HA (1∶100) in TBS containing 1% bovine serum albumin, then incubated with Alexa Fluor 594-conjugated anti-mouse immunoglobulin G (1∶100). To stain the nucleus, cells were cultured with DAPI (1∶10000). The stained cells were mounted with ProLong Gold antifade reagent (Invitrogen) on glass slides and analyzed using a fluorescence microscope (FV1000-D IX81; Olympus).

### Western blot analysis

293T cells were lysis buffer containing 1% Nonidet P-40, 150 mM NaCl, 20 mM Tris-HCl, 1 mM EDTA, pH 7.5, and protease inhibitor cocktail (Roche). The cell lysates were separated by SDS-PAGE and transferred to PVDF membranes, and subjected to Western blot analysis using the indicated antibodies as described previously [59].

### Isolation of nuclear fractions

Cells were treated with indicated stimulants. Treated cells were washed with PBS, lysed by Buffer A (10 mM HEPES-KOH (pH 7.8), 10 mM KCl, 0.1 mM EDTA (pH 8.0), 0.1% Nonidet P-40) and incubated on ice for 5 min. Lysates were centrifuged at 5000 r.p.m. for 5 min, then the pellets were re-suspended by Buffer C (50 mM HEPES-KOH (pH 7.8), 420 mM KCl, 0.1 mM EDTA (pH 8.0), 5 mM MgCl_2_, 20% Glycerol) and protease inhibitor cocktail (Roche) and incubated on ice for 30 min. To isolate nuclear fractions, lysates were centrifuged at 14000 r.p.m. for 10 min. The resulting supernatants contained the nuclear fractions.

### Statistical analysis

We used unpaired student's t-test and log-rank test to determine statistical significance among experimental data.

## Supporting Information

Figure S1 **Targeted disruption of the murine** ***CREBH*** **gene.** (A) The structure of the murine *CREBH* gene, the targeting vector and the predicted disrupted gene. Open boxes denote the coding exon. H, *HincII*; B, *BamHI*. (B) Southern blot analysis of offspring from the heterozygote intercrosses. Genomic DNA was extracted from mouse tails, digested with *HincII* and *BamHI*, separated by electrophoresis and hybridized with the radiolabelled probe indicated in (A). Southern blotting gave a single 2.5-kb band for wild-type (+/+), a 1.7-kb band for homozygous (−/−) and both bands for heterozygous (+/−) mice. (C) Northern blot analysis of liver cells taken from wild-type and CREBH-deficient mice. Total RNA (15 µg) extracted from livers was separated by electrophoresis, transferred to nylon membrane and hybridized using the CREBH fragment as a probe. The same membrane was rehybridized with a β-actin probe.
(TIF)

Figure S2 **Illustration of the metabolite of sulpyrine.** Sulpyrine is non-enzymatically hydrolysed to 4-methylaminoantipyrine (4-MAA), which is further metabolized to 4-aminoantipyrine (4-AA), and 4-formylaminoantipyrine (4-FAA) in the liver.
(TIF)

Figure S3 **HPLC chromatograms for the identification of 4-AA and 4-FAA.** (A) Representative HPLC chromatograms of 4-AA standard (left) and 4-FAA standard (right). (B) A representative HPLC chromatogram of serum sample of sulpyrine-administrated mouse. Data are representative of three (A, B) independent experiments.
(TIF)

Figure S4 **Almost 1000 ng/ul 4-AA in the sera is the threshold of live-and-dead.** (A) Wild-type mice were intraperitoneally injected with 1.1 mg/g of 4-AA. Sera of alive group (n = 4) and dead group (n = 5) were taken at indicated time points. Serum concentration of 4-AA were measured by a HPLC assay. The assay was performed on mice only when they were alive. N.D., not detected. ***, P<0.001. (B) Wild-type (n = 5) and CREBH-deficient (n = 5) mice were intraperitoneally injected with 1.1 mg/g of 4-AA and 4-FAA. Survival rates were monitored for 180 min and 48 hr. Data are representative of two (A, B) independent experiments.
(TIF)

Figure S5 ***In vivo*** **transfection of human CREBH.** (A, B) CREBH-deficient mice were transfected with HA-tagged hCREBH expression vectors (n = 4) or empty vectors (n = 4). At 24 hr after transfection, livers were taken from these mice. Gene expression of hCREBH was analysed by a quantitative RT-PCR and an Immunofluorescence assay. The sections were stained with anti-HA mouse antibody, then stained with Alexa Fluor 488-conjugated anti-mouse IgG (green) together with DAPI (blue). Stained cells were analysed using a confocal microscope. Bars, 100 µm. ***, P<0.001. Data are representative of two (A, B) independent experiments.
(TIF)

Figure S6 ***In vivo*** **transfection of human CYP2B6.** (A, B) CREBH-deficient mice were transfected with HA-tagged hCYP2B6 expression vectors (n = 4) or empty vectors (n = 4). At 24 hr after transfection, livers were taken from these mice. Gene expression of hCYP2B6 was analysed by a quantitative RT-PCR and an Immunofluorescence assay. The sections were stained with anti-HA mouse antibody, then stained with Alexa Fluor 488-conjugated anti-mouse IgG (green) together with DAPI (blue). Stained cells were analysed using a confocal microscope. Bars, 100 µm. ***, P<0.001. Data are representative of two (A, B) independent experiments.
(TIF)

Figure S7 **CREBH and CAR independently activate CYP2B10 promoter.** (A) Illustration of luciferase reporter plasmids containing the CYP2B10 promoter with or without the CAR responsive element called Nr1 region. (B) Huh7 cells were transiently transfected with luciferase reporter plasmids containing the CYP2B10 promoter with or without Nr1 region together with control or hCREBH-F expression vector. Relative luciferase activities were shown as fold increases over the background levels shown by lysates prepared from control vector-transfected cells. Error bars are means ± S.D. of triplicates. N.S., not significant. ***, P<0.001. (C) Hepatocytes of Wild-type mice and CREBH-deficient mice were taken by a perfusion apparatus. Hepatocytes were transiently transfected with luciferase reporter plasmids containing the CYP2B10 promoter with Nr1 region together with control or hCAR expression vector. Relative luciferase activities were shown as fold increases over the background levels shown by lysates prepared from control vector-transfected cells. Error bars are means ± S.D. of five ways. N.S., not significant. **, P<0.01; ***, P<0.001. Data are representative of three (B, C) independent experiments.
(TIF)

Figure S8 **RNAi vector for CREBH suppressed the expression of CREBH** ***in vitro*** **.** 293T cells were transfected with 2 µg HA-tagged mCREBH-F expression vectors together with indicated volumes of RNAi vectors targeting for mCREBH. At 24 hr after transfection, whole-cell lysates were prepared, and the knockdown efficiency of RNAi vectors for mCREBH was analysed by western blot analysis using anti-HA antibody. Data are representative of two independent experiments.
(TIF)

Table S1 **A list of the sulpyrine-inducible genes studied.**
(TIFF)

Table S2 **Primers list used in this study.**
(TIFF)
