# Supplementary figures and images for: CREBH Determines the Severity of Sulpyrine-Induced Fatal Shock
Source: PLoS One. 2013 Feb 7;8(2):e55800. doi: 10.1371/journal.pone.0055800 (PMC3567110; doi:10.1371/journal.pone.0055800)

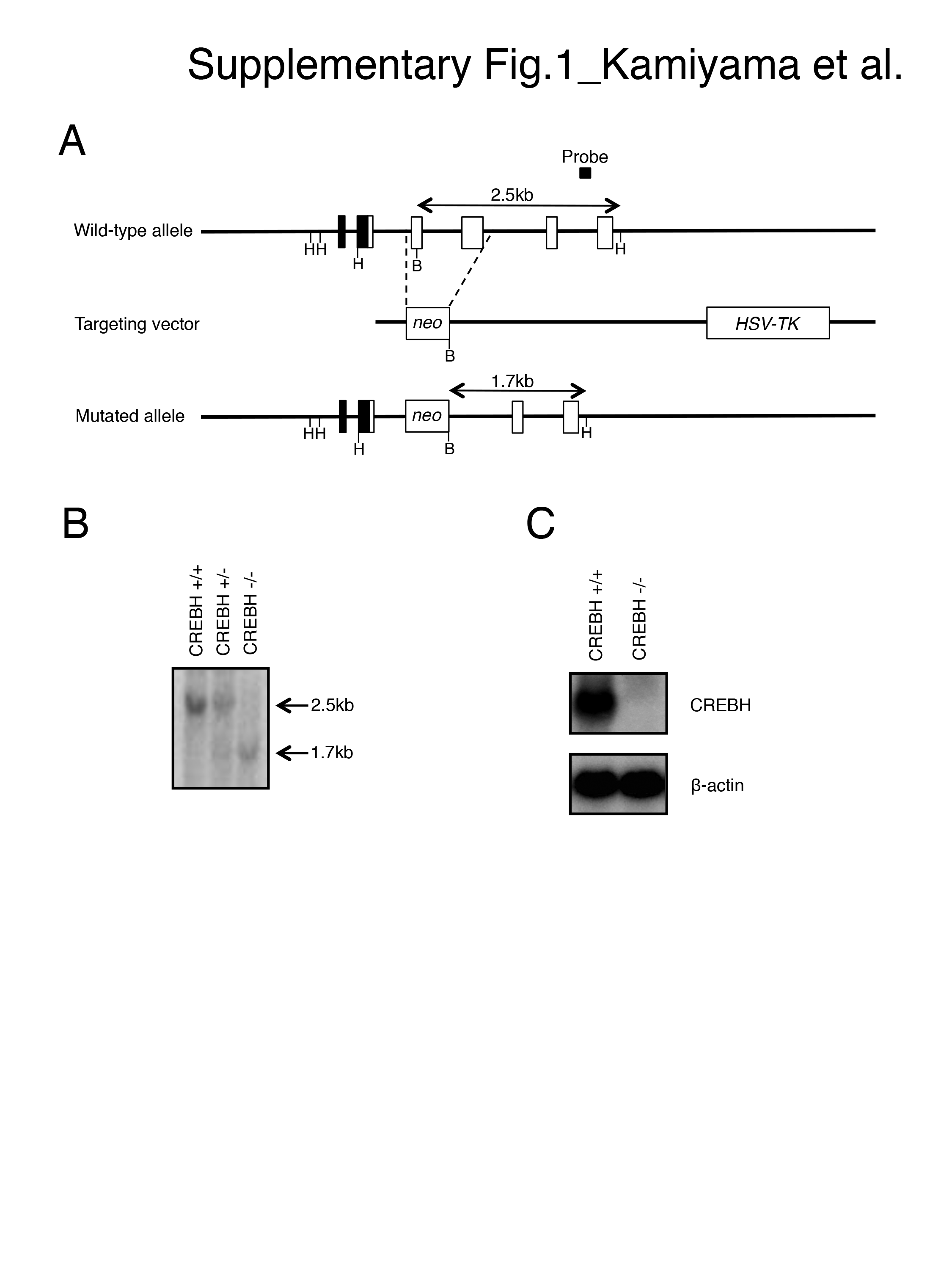

Supplement: Figure S1 — Targeted disruption of the murine CREBH gene. (A) The structure of the murine CREBH gene, the targeting vector and the predicted disrupted gene. Open boxes denote the coding exon. H, HincII; B, BamHI. (B) Southern blot analysis of offspring from the heterozygote intercrosses. Genomic DNA was extracted from mouse tails, digested with HincII and BamHI, separated by electrophoresis and hybridized with the radiolabelled probe indicated in (A). Southern blotting gave a single 2.5-kb band for wild-type (+/+), a 1.7-kb band for homozygous (−/−) and both bands for heterozygous (+/−) mice. (C) Northern blot analysis of liver cells taken from wild-type and CREBH-deficient mice. Total RNA (15 µg) extracted from livers was separated by electrophoresis, transferred to nylon membrane and hybridized using the CREBH fragment as a probe. The same membrane was rehybridized with a β-actin probe. (TIF) [file pone.0055800.s001.tif]

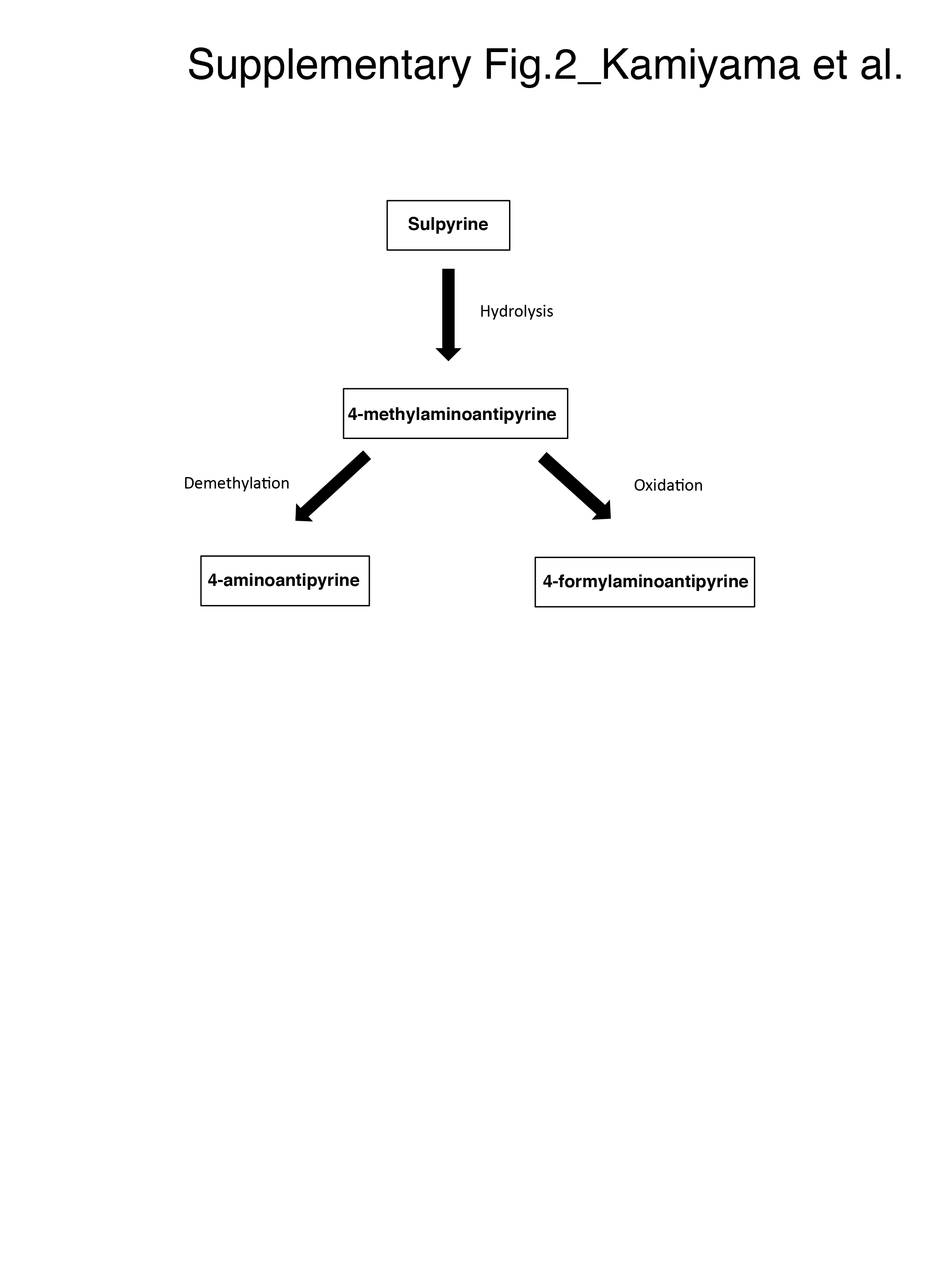

Supplement: Figure S2 — Illustration of the metabolite of sulpyrine. Sulpyrine is non-enzymatically hydrolysed to 4-methylaminoantipyrine (4-MAA), which is further metabolized to 4-aminoantipyrine (4-AA), and 4-formylaminoantipyrine (4-FAA) in the liver. (TIF) [file pone.0055800.s002.tif]

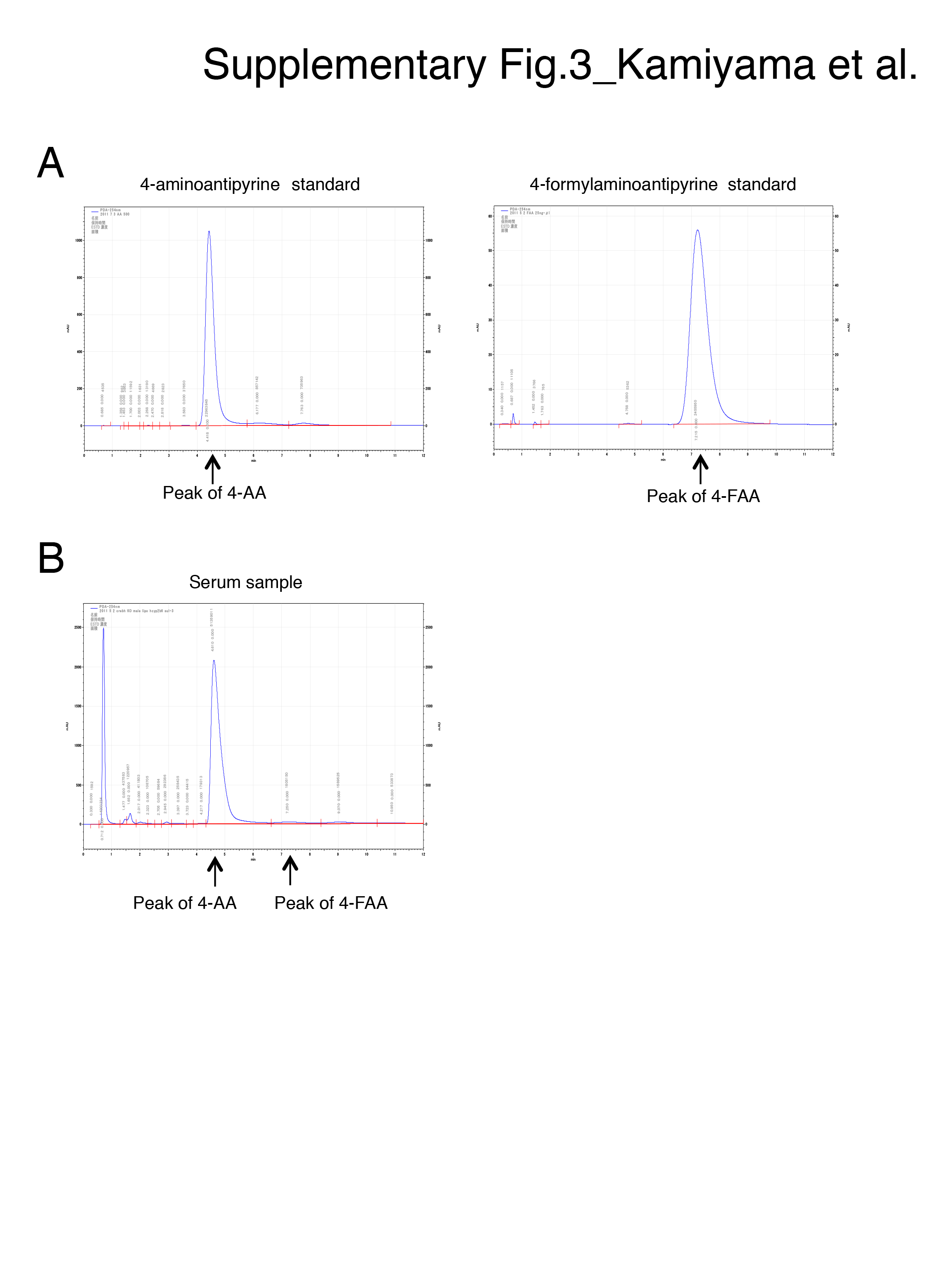

Supplement: Figure S3 — HPLC chromatograms for the identification of 4-AA and 4-FAA. (A) Representative HPLC chromatograms of 4-AA standard (left) and 4-FAA standard (right). (B) A representative HPLC chromatogram of serum sample of sulpyrine-administrated mouse. Data are representative of three (A, B) independent experiments. (TIF) [file pone.0055800.s003.tif]

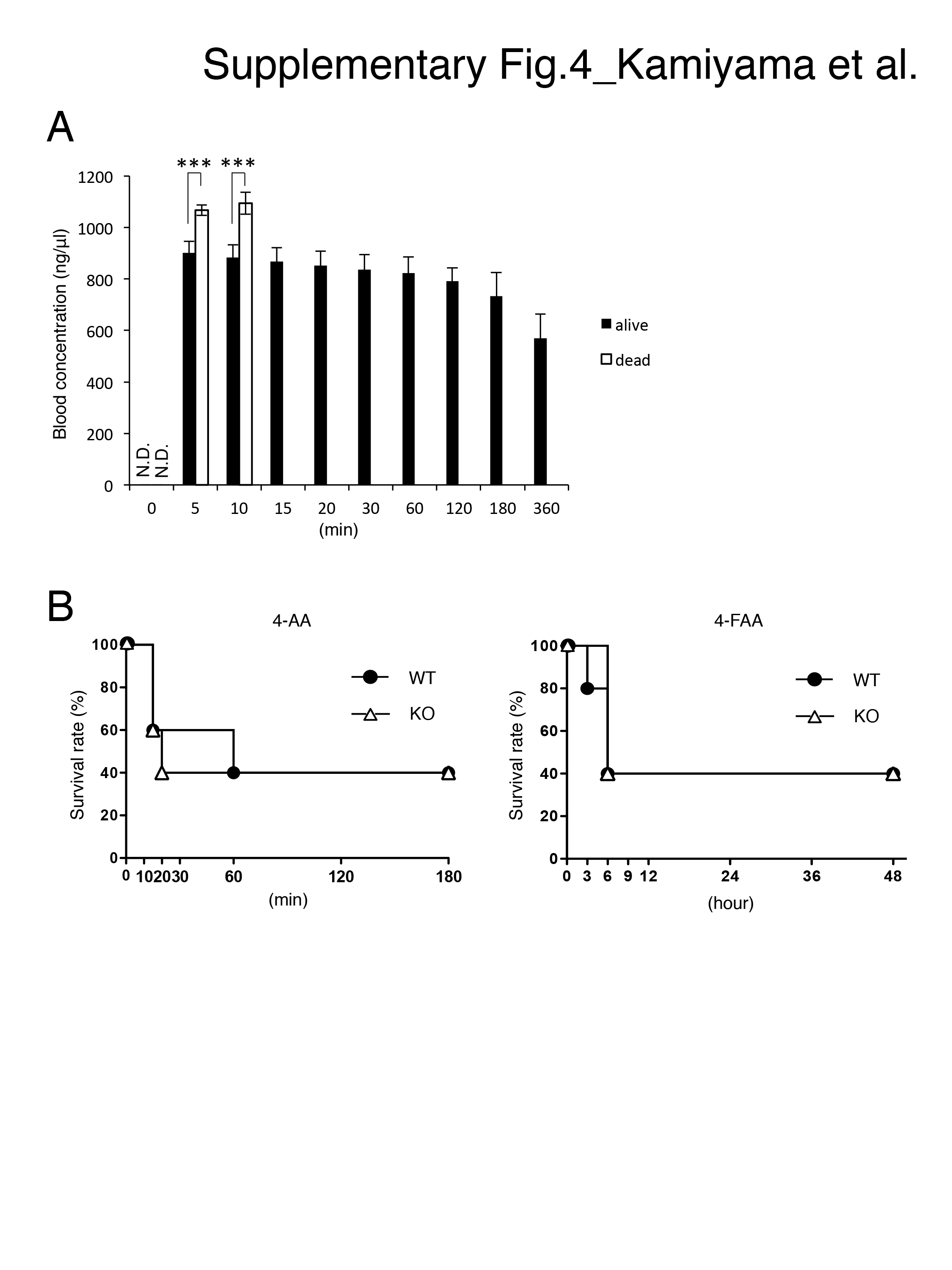

Supplement: Figure S4 — Almost 1000 ng/ul 4-AA in the sera is the threshold of live-and-dead. (A) Wild-type mice were intraperitoneally injected with 1.1 mg/g of 4-AA. Sera of alive group (n = 4) and dead group (n = 5) were taken at indicated time points. Serum concentration of 4-AA were measured by a HPLC assay. The assay was performed on mice only when they were alive. N.D., not detected. ***, P<0.001. (B) Wild-type (n = 5) and CREBH-deficient (n = 5) mice were intraperitoneally injected with 1.1 mg/g of 4-AA and 4-FAA. Survival rates were monitored for 180 min and 48 hr. Data are representative of two (A, B) independent experiments. (TIF) [file pone.0055800.s004.tif]

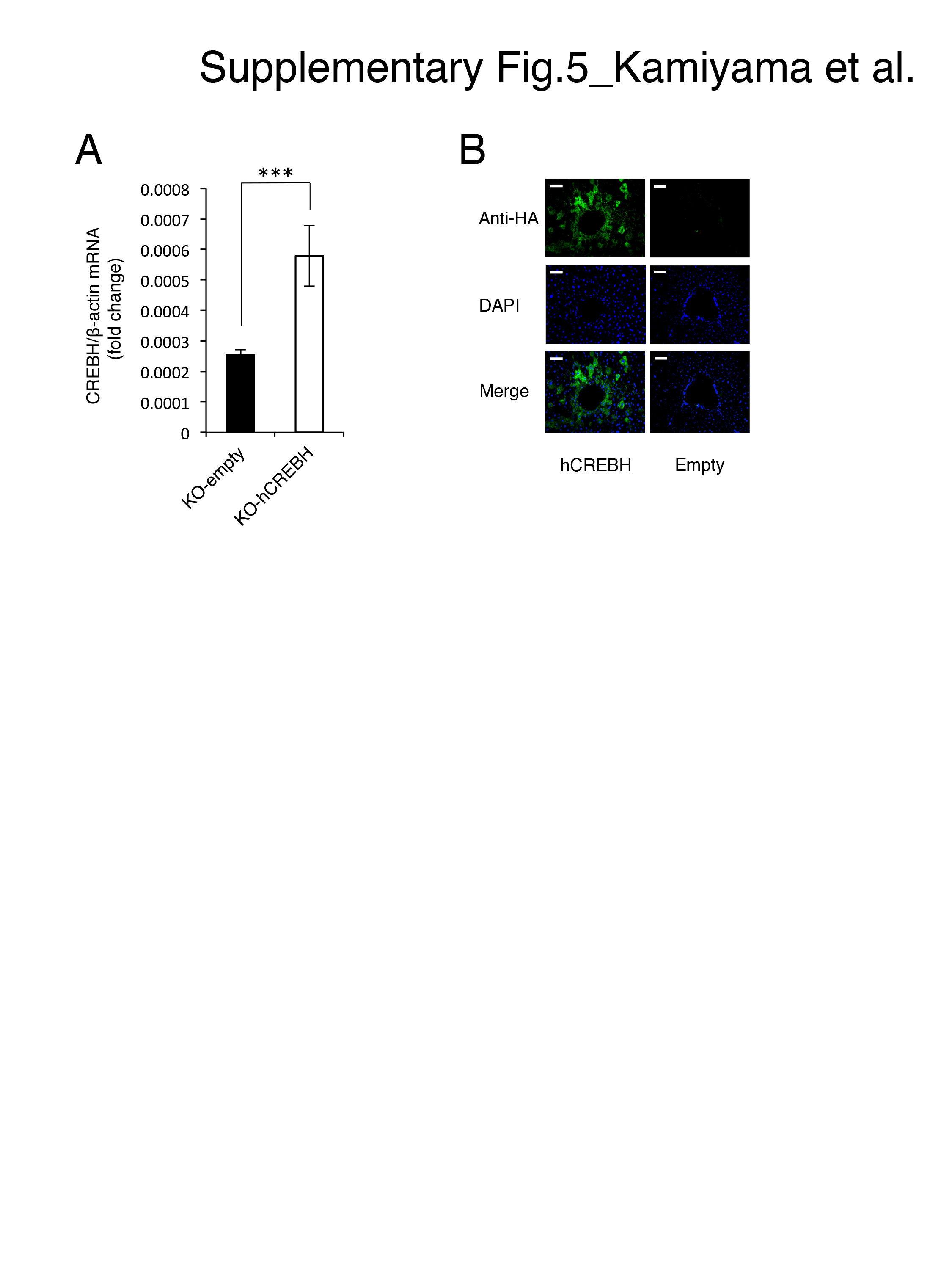

Supplement: Figure S5 — In vivo transfection of human CREBH. (A, B) CREBH-deficient mice were transfected with HA-tagged hCREBH expression vectors (n = 4) or empty vectors (n = 4). At 24 hr after transfection, livers were taken from these mice. Gene expression of hCREBH was analysed by a quantitative RT-PCR and an Immunofluorescence assay. The sections were stained with anti-HA mouse antibody, then stained with Alexa Fluor 488-conjugated anti-mouse IgG (green) together with DAPI (blue). Stained cells were analysed using a confocal microscope. Bars, 100 µm. ***, P<0.001. Data are representative of two (A, B) independent experiments. (TIF) [file pone.0055800.s005.tif]

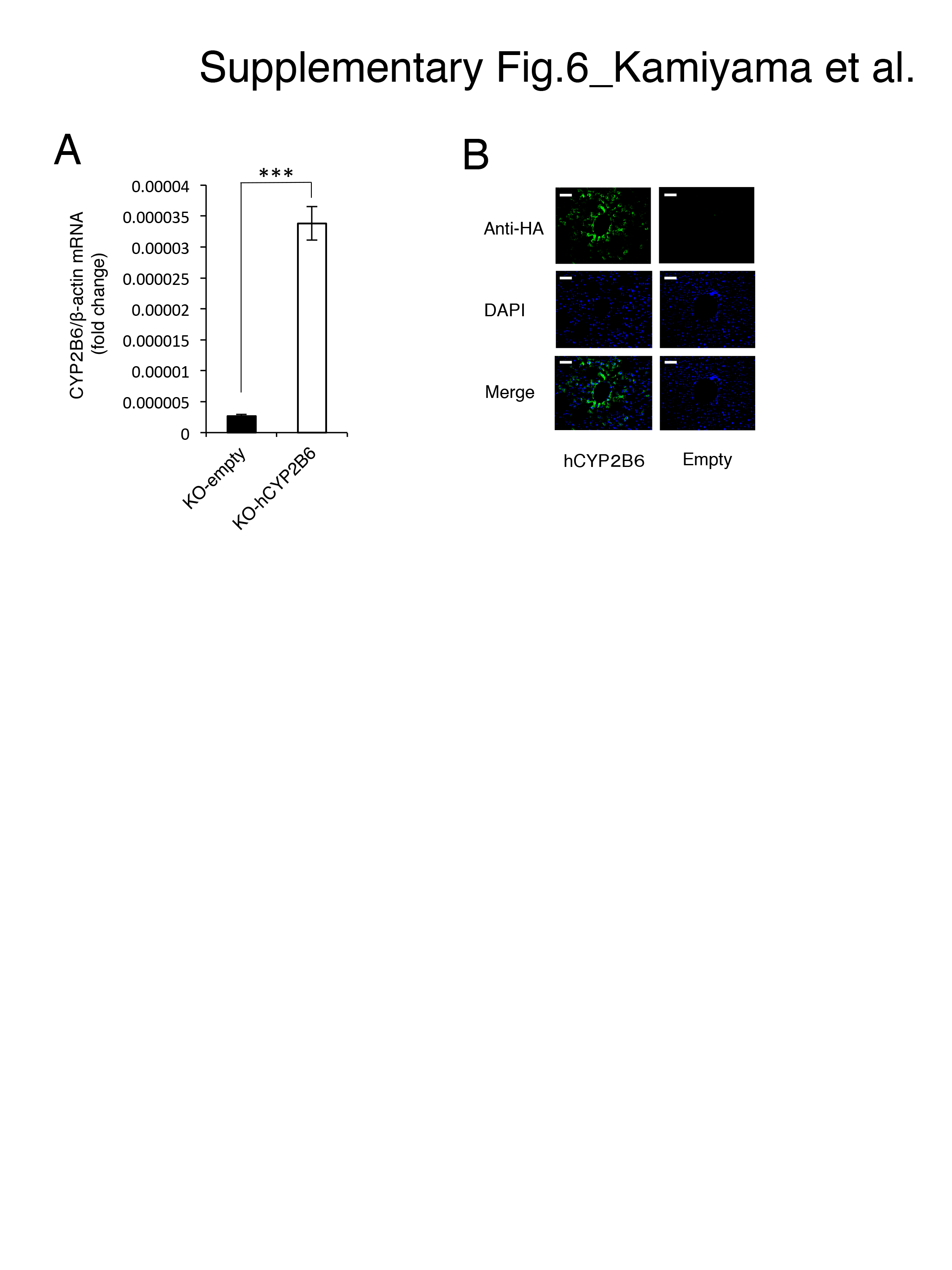

Supplement: Figure S6 — In vivo transfection of human CYP2B6. (A, B) CREBH-deficient mice were transfected with HA-tagged hCYP2B6 expression vectors (n = 4) or empty vectors (n = 4). At 24 hr after transfection, livers were taken from these mice. Gene expression of hCYP2B6 was analysed by a quantitative RT-PCR and an Immunofluorescence assay. The sections were stained with anti-HA mouse antibody, then stained with Alexa Fluor 488-conjugated anti-mouse IgG (green) together with DAPI (blue). Stained cells were analysed using a confocal microscope. Bars, 100 µm. ***, P<0.001. Data are representative of two (A, B) independent experiments. (TIF) [file pone.0055800.s006.tif]

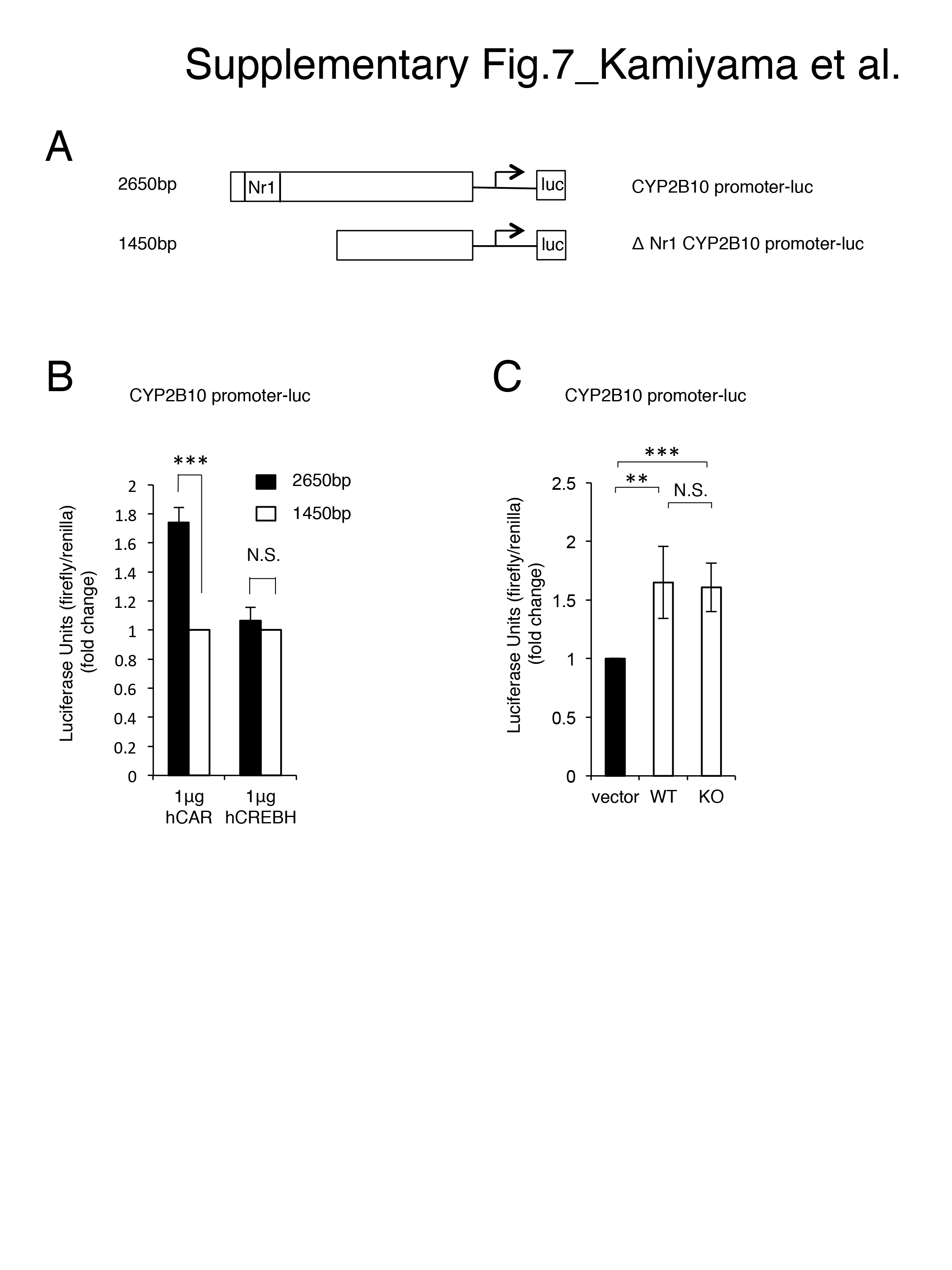

Supplement: Figure S7 — CREBH and CAR independently activate CYP2B10 promoter. (A) Illustration of luciferase reporter plasmids containing the CYP2B10 promoter with or without the CAR responsive element called Nr1 region. (B) Huh7 cells were transiently transfected with luciferase reporter plasmids containing the CYP2B10 promoter with or without Nr1 region together with control or hCREBH-F expression vector. Relative luciferase activities were shown as fold increases over the background levels shown by lysates prepared from control vector-transfected cells. Error bars are means ± S.D. of triplicates. N.S., not significant. ***, P<0.001. (C) Hepatocytes of Wild-type mice and CREBH-deficient mice were taken by a perfusion apparatus. Hepatocytes were transiently transfected with luciferase reporter plasmids containing the CYP2B10 promoter with Nr1 region together with control or hCAR expression vector. Relative luciferase activities were shown as fold increases over the background levels shown by lysates prepared from control vector-transfected cells. Error bars are means ± S.D. of five ways. N.S., not significant. **, P<0.01; ***, P<0.001. Data are representative of three (B, C) independent experiments. (TIF) [file pone.0055800.s007.tif]

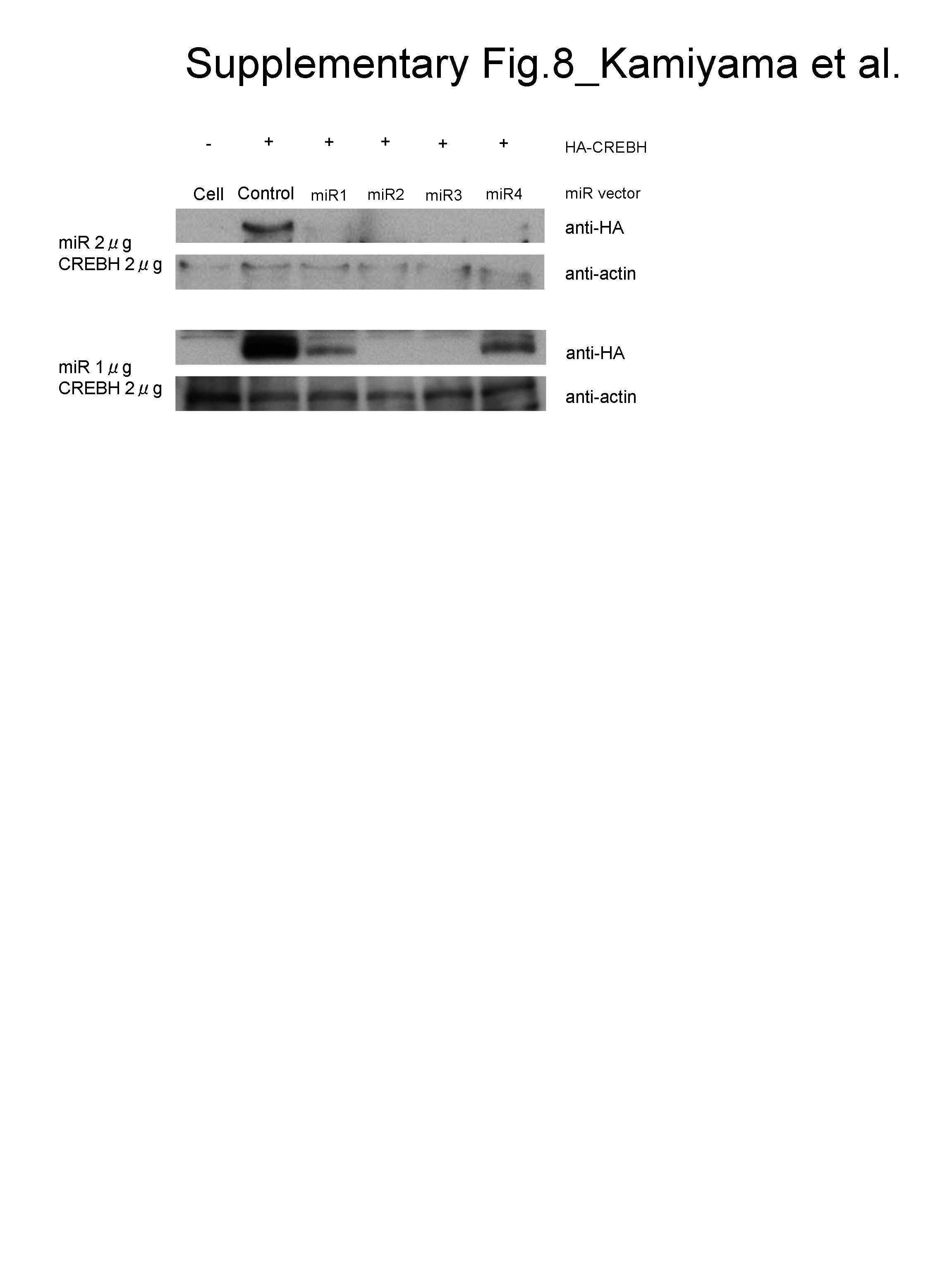

Supplement: Figure S8 — RNAi vector for CREBH suppressed the expression of CREBH in vitro . 293T cells were transfected with 2 µg HA-tagged mCREBH-F expression vectors together with indicated volumes of RNAi vectors targeting for mCREBH. At 24 hr after transfection, whole-cell lysates were prepared, and the knockdown efficiency of RNAi vectors for mCREBH was analysed by western blot analysis using anti-HA antibody. Data are representative of two independent experiments. (TIF) [file pone.0055800.s008.tif]

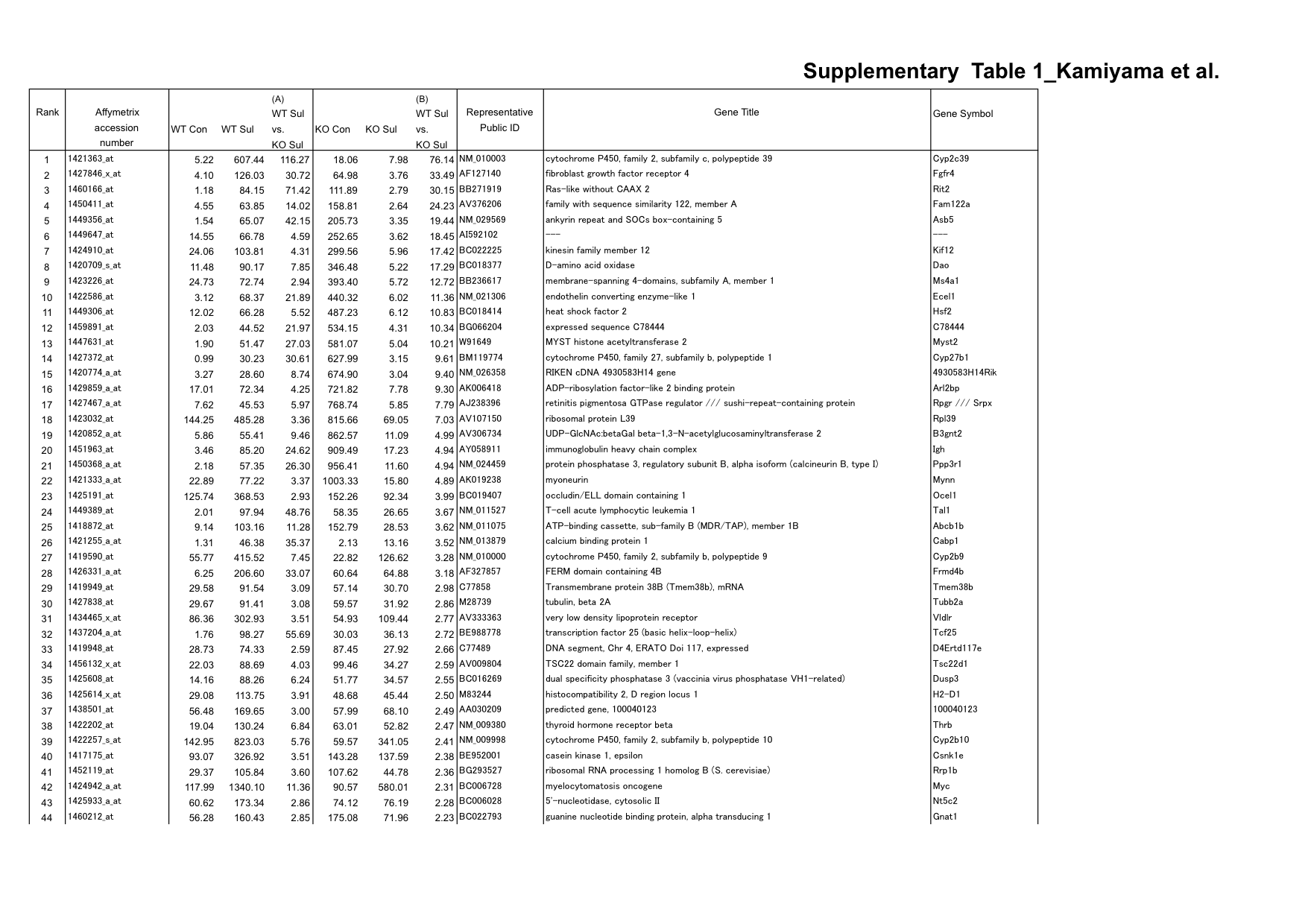

Supplement: Table S1 — A list of the sulpyrine-inducible genes studied. (TIFF) [file pone.0055800.s009.tiff]

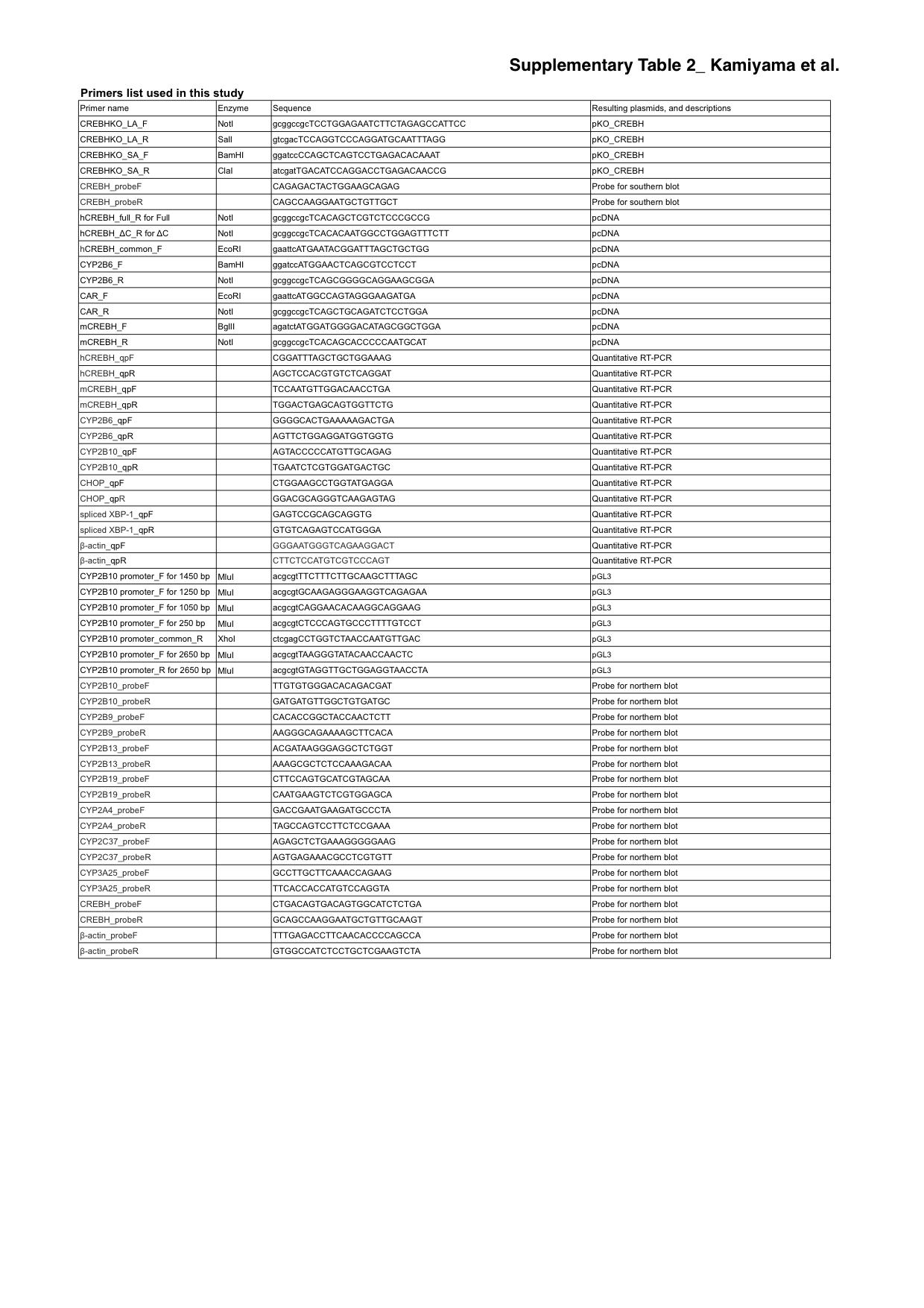

Supplement: Table S2 — Primers list used in this study. (TIFF) [file pone.0055800.s010.tiff]
